# The astrocytic TRPA1 channel mediates an intrinsic protective response to vascular cognitive impairment via LIF production

**DOI:** 10.1126/sciadv.adh0102

**Published:** 2023-07-21

**Authors:** Masashi Kakae, Hiroki Nakajima, Shota Tobori, Ayaka Kawashita, Jun Miyanohara, Misa Morishima, Kazuki Nagayasu, Takayuki Nakagawa, Eiji Shigetomi, Schuichi Koizumi, Yasuo Mori, Shuji Kaneko, Hisashi Shirakawa

**Affiliations:** ^1^ Department of Molecular Pharmacology, Graduate School of Pharmaceutical Sciences, Kyoto University, Kyoto, Japan.; ^2^ Department of Clinical Pharmacology and Pharmacotherapy, School of Pharmaceutical Sciences, Wakayama Medical University, Wakayama, Japan.; ^3^ Department of Clinical Pharmacology and Therapeutics, Kyoto University Hospital, Kyoto, Japan.; ^4^ Department of Neuropharmacology, Interdisciplinary Graduate School of Medicine, University of Yamanashi, Yamanashi, Japan.; ^5^ Yamanashi GLIA Center, University of Yamanashi, Yamanashi, Japan.; ^6^ Department of Synthetic Chemistry and Biological Chemistry, Graduate School of Engineering, Kyoto University, Kyoto, Japan.

## Abstract

Vascular cognitive impairment (VCI) refers to cognitive alterations caused by vascular disease, which is associated with various types of dementia. Because chronic cerebral hypoperfusion (CCH) induces VCI, we used bilateral common carotid artery stenosis (BCAS) mice as a CCH-induced VCI model. Transient receptor potential ankyrin 1 (TRPA1), the most redox-sensitive TRP channel, is functionally expressed in the brain. Here, we investigated the pathophysiological role of TRPA1 in CCH-induced VCI. During early-stage CCH, cognitive impairment and white matter injury were induced by BCAS in TRPA1-knockout but not wild-type mice. TRPA1 stimulation with cinnamaldehyde ameliorated BCAS-induced outcomes. RNA sequencing analysis revealed that BCAS increased leukemia inhibitory factor (LIF) in astrocytes. Moreover, hydrogen peroxide–treated TRPA1-stimulated primary astrocyte cultures expressed LIF, and culture medium derived from these cells promoted oligodendrocyte precursor cell myelination. Overall, TRPA1 in astrocytes prevents CCH-induced VCI through LIF production. Therefore, TRPA1 stimulation may be a promising therapeutic approach for VCI.

## Teaser

TRPA1-mediated LIF production is a promising target for vascular cognitive impairment, linked to various types of dementia.

## INTRODUCTION

Vascular cognitive impairment (VCI) is a syndrome defined as cognitive decline caused by vascular disease (*1*). Chronic cerebral hypoperfusion (CCH), which is elicited by aging, metabolic syndromes, atherosclerosis, hypertension, obesity (*2*), and hypotension (*3*), is commonly present in various types of dementia (*4*–*6*), and CCH-associated small vessel disease (SVD) is the major contributor to VCI (*7*, *8*). Growing evidence suggests that CCH is the cause of white matter lesions, which are a key character of VCI. White matter lesions that precede neuronal dysfunction have been observed in a CCH-induced VCI mouse model (*9*, *10*). Furthermore, patients with various types of dementia show white matter lesions (*11*). Thus, although the importance of CCH-induced white matter lesions in VCI has received widespread attention, effective therapeutic targets for VCI are still under development because the precise molecular mechanisms that detect and regulate pathological changes in VCI remain to be elucidated.

Transient receptor potential (TRP) channels are biosensors that detect changes of the surrounding environment and cellular redox status (*12*, *13*), and we have previously reported that certain TRP channels participate in the development of various disorders (*9*, *14*–*16*). Among TRP channels, TRP ankyrin 1 (TRPA1) was initially found to be a noxious cold-activated channel (*17*). It is expressed in a subset of nociceptive sensory neurons in peripheral nerves and is related to various types of pain generation (*18*). In the central nervous system (CNS), TRPA1 is localized in non-neuronal cells such as astrocytes (*19*–*21*), cerebral endothelial cells (*22*), and oligodendrocytes (*23*). Studies have reported that TRPA1 in these cells plays key roles in cerebrovascular diseases and dementia, such as cerebral ischemia (*23*, *24*) and Alzheimer’s disease (AD) (*25*, *26*); however, the contribution of TRPA1 to pathology is controversial, as it has both protective and destructive effects. Collectively, TRPA1 seems to function as a polymodal sensor in vascular diseases, and clarification of TRPA1-mediating molecular mechanism could lead to a promising approach for VCI therapy.

In this study, we used a bilateral common carotid artery stenosis (BCAS) mouse as a mouse model of CCH-induced VCI, which shows a decrease in the myelin sheath preceding neuronal dysfunction (*9*, *10*, *27*), similar to the human clinical picture (*28*). Around postoperative day 14, BCAS-operated mice do not show cognitive decline but often show a mild reduction in myelinated fiber density and a slight deficit in myelin integrity (*9*). Subsequently around postoperative day 28 after BCAS, a marked reduction in myelin sheath, “white matter damage,” and cognitive dysfunction are observed (*9*, *27*). To clarify the involvement of TRPA1 in CCH-induced VCI, we used genetically engineered mice: TRPA1-knockout (TRPA1-KO) and cell-specific conditional TRPA1-KO mice. We show that TRPA1 deficiency accelerates BCAS-induced cognitive impairment and white matter injury and that the increase of leukemia inhibitory factor (LIF) through TRPA1 stimulation in astrocytes plays a protective role in CCH-induced VCI. Our findings reveal an intrinsic protective mechanism for the maintenance of white matter integrity in VCI.

## RESULTS

### TRPA1 deficiency accelerates BCAS-induced cognitive impairment and white matter injury

To investigate the involvement of TRPA1 in VCI, we assessed *Trpa1* mRNA expression with quantitative reverse transcription polymerase chain reaction (RT-PCR). After BCAS, *Trpa1* mRNA expression in the corpus callosum was not yet increased on postoperative day 14, but was increased on postoperative day 28 (fig. S1, A to C).

**Fig. 1.**
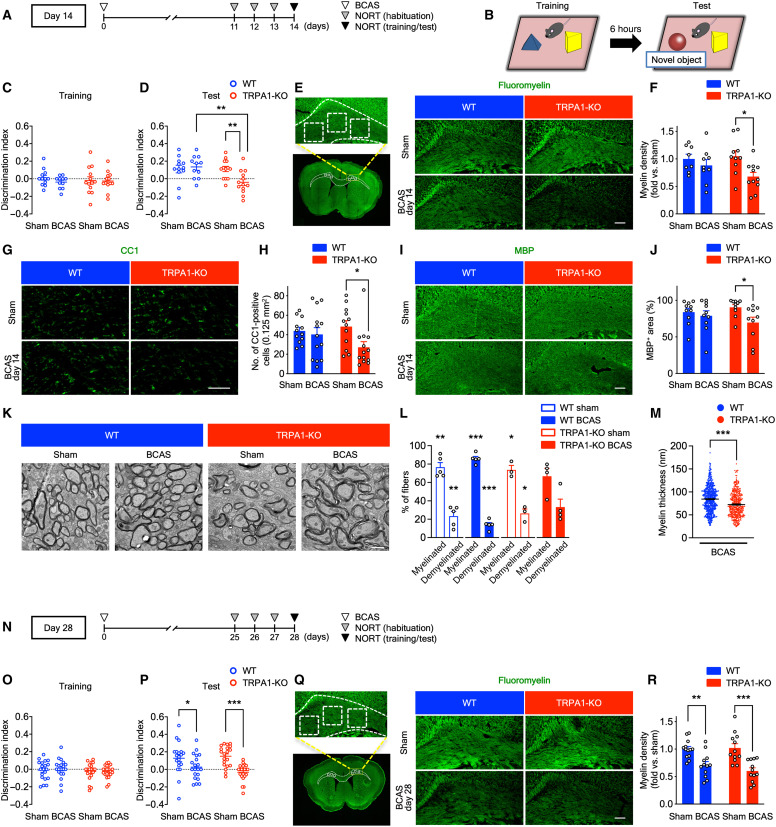
BCAS induces cognitive impairment and white matter injury on day 14 in TRPA1-KO but not WT mice. (**A**, **B**, and **N**) The experimental time course on days 14 (A) and 28 (N) and a schematic (B) for the NORT. (**C**, **D**, **O**, and **P**) Discrimination indexes for exploring the blue quadrangular object during the training session (C) and (O) and the wooden ball, i.e., the “novel object,” during the test session (D) and (P) on days 14 (C) and (D) and 28 (O) and (P). (**E** to **M**, **Q**, and **R**) Representative images of myelin staining (E) and (Q), immunostaining with anti-CC1 (G) or MBP (I) antibody, and TEM (K) in the corpus callosum as well as summarized data for relative myelin density (F) and (R), the number of CC1-positive cells (H), the percentage of MBP-positive surface areas (J) and myelinated and demyelinated fibers (L), and myelin thickness (M) on days 14 (E) to (M) and 28 (Q) and (R). The dashed line–surrounded area is the corpus callosum and the square-surrounded areas are quantified regions in myelin staining (E) and (Q). Values are means ± SEM. Scale bars, 1 μm (K) and 100 μm (E), (G), (I), and (Q). (C) and (D) *n* = 11 to 13; (F) *n* = 8 to 11; (H) *n* = 12 to 13; (J) *n* = 10; (L) *n* = 3 to 5; (M) *n* = 4 to 5 mice; (O) and (P) *n* = 20 to 22; (R) *n* = 12 to 13. *P < 0.05, **P < 0.01, and ***P < 0.001 for two-way analysis of variance (ANOVA) with Bonferroni’s post hoc test (D), (F), (H), (J), (P), and (R). *P < 0.05, **P < 0.01, and ***P < 0.001 for one sample *t* test (L). ***P < 0.001 for two-tailed unpaired Student’s *t* test (M).

Next, we assessed cognitive impairment and white matter damage on postoperative days 14 and 28 in wild-type (WT) and TRPA1-KO mice (Fig. 1). BCAS successfully decreased the regional cerebral blood flow to ~65% compared with the baseline at 60 min after operation. This change did not differ between WT and TRPA1-KO mice (fig. S1D). To assess cognitive function, we performed the novel object recognition test (NORT) (Fig. 1, A and B). On day 14, which represents early-stage CCH, the discrimination index (DI) for exploring novel objects did not differ between the groups during the training session (Fig. 1C). However, the DI was significantly decreased in BCAS-operated TRPA1-KO mice compared with sham-operated TRPA1-KO mice. By contrast, such decreases in DI were not observed in BCAS-operated WT mice compared with sham-operated WT mice (Fig. 1D). Total exploratory times for the two objects did not differ between the groups, demonstrating similar exploratory behavior (fig. S2, A and B). Because white matter injury is a characteristic of VCI (*9*, *10*), we performed myelin staining with FluoroMyelin in the corpus callosum after BCAS to evaluate VCI-related white matter injury. Consistent with cognitive impairment, the myelin density was significantly decreased in BCAS-operated TRPA1-KO mice compared with sham-operated TRPA1-KO mice. By contrast, the myelin density did not differ between sham- and BCAS-operated WT mice on day 14 (Fig. 1, E and F). To further assess white matter damage, we performed immunostaining of CC1 (a marker of oligodendrocytes) and myelin basic protein (MBP; a marker for mature oligodendrocytes). We found that, similar to myelin staining, the number of CC1-positive cells and MBP-positive area in the corpus callosum were decreased in BCAS-operated TRPA1-KO mice. By contrast, no differences were observed in WT mice on day 14 (Fig. 1, G to J). For further investigation, we assessed myelin structure using transmission electron microscopy (TEM). We found that the increase in abnormal myelin structures and demyelinated fibers in BCAS-operated TRPA1-KO mice and no differences in the percentage of myelinated and demyelinated fibers were observed in BCAS-operated TRPA1-KO mice on day 14 (Fig. 1, K and L). Moreover, we detected the decrease in myelin thickness in BCAS-operated TRPA1-KO mice compared with BCAS-operated WT mice on day 14 (Fig. 1M). In addition, we performed immunostaining of oligodendrocyte transcription factor 2 (OLIG2), SRY-related HMG-box 10 (SOX10) (a marker for oligodendrocyte lineage cells), and platelet-derived growth factor receptor α [PDGFRα; a marker for oligodendrocyte precursor cells (OPCs)] to further investigate the involvement of oligodendrocyte lineage cells. We found that there were no differences in the number of oligodendrocyte lineage cells and OPCs although the number of OLIG2-positive cells tended to be decreased in BCAS-operated TRPA1-KO mice similar to immunostaining of CC1 and MBP (fig. S2, C to H). These results suggest that TRPA1 deficiency accelerates CCH-induced cognitive impairment and loss of mature oligodendrocytes but not immature oligodendrocyte lineage cells such as OPCs.

On day 28, which represents middle- to late-stage CCH (Fig. 1N), DI did not differ between the groups during the training session (Fig. 1O). By contrast, DI was significantly decreased in BCAS-operated mice compared with sham-operated mice in both WT and TRPA1-KO mice during the test session on day 28 (Fig. 1P). Exploratory times for the two objects were similar across the groups (fig. S2, I and J). Consistent with cognitive impairment, the myelin density was significantly decreased in BCAS-operated WT and TRPA1-KO mice compared with sham-operated mice on day 28 (Fig. 1, Q and R).

Next, we performed the novel location recognition test to assess spatial memory function (fig. S3, A to E). On day 14, DI did not differ between the groups, and all groups showed a preference for the relocated object (fig. S3, B and C). Similar to day 14, DI did not differ between the groups on day 28 (fig. S3, D and E). Moreover, to assess neuronal survival, we performed immunostaining with NeuN, a marker for neurons, which play an important role in cognitive function, including spatial memory. The numbers of NeuN-positive cells in the CA1, CA3, and dentate gyrus were unchanged in all groups on day 28 (fig. S3, F to I). These results are consistent with a previous study (*9*) and suggest that CCH for 28 days does not induce spatial memory dysfunction or neuronal damage in the hippocampus. Together, our results indicate that TRPA1 deficiency accelerates CCH-induced VCI and white matter injury from early-stage CCH, on day 14, when BCAS-operated WT mice do not show cognitive impairment and marked white matter injury.

### TRPA1 stimulation suppresses BCAS-induced cognitive impairment and white matter injury

**Fig. 2.**
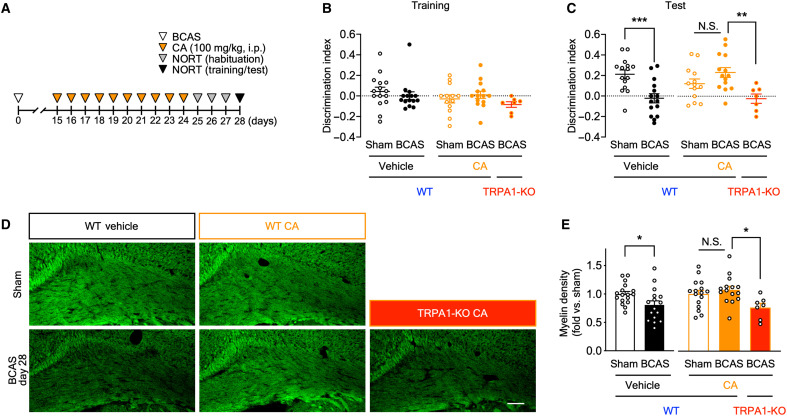
Cinnamaldehyde (CA), a TRPA1 agonist, attenuates BCAS-induced cognitive impairment and white matter injury. (**A**) Experimental time course for CA administration and the NORT. (**B** and **C**) Discrimination indexes for exploring the blue quadrangular object during the training session (B) and the wooden ball, i.e., novel object, during the test session (C) on postoperative day 28. (**D** and **E**) Representative images of myelin staining in the corpus callosum (D) and summarized data for relative myelin density (E) on postoperative day 28. Values are means ± SEM. Scale bar, 100 μm. (B) and (C) *n* = 15 (vehicle), *n* = 7 to 14 (CA); (E) *n* = 16 to 17 (vehicle), *n* = 7 to 16 (CA). *P < 0.05 and ***P < 0.001 for two-tailed unpaired Student’s *t* test [(C) vehicle and (E) vehicle]. *P < 0.05 and **P < 0.01 for one-way ANOVA with Tukey’s post hoc test [(C) CA and (E) CA]. i.p., intraperitoneal administration; N.S., not significant.

To investigate the effects of TRPA1 stimulation, we intraperitoneally administered WT mice with cinnamaldehyde, a TRPA1 agonist, on postoperative days 15 to 24 (Fig. 2A). DI did not differ between the groups during the NORT training session (Fig. 2B). During the test session, DI was significantly decreased in vehicle-administered BCAS-operated WT mice but was unchanged in cinnamaldehyde-administered BCAS-operated WT mice. Notably, the inhibitory effect of cinnamaldehyde was diminished in TRPA1-KO mice (Fig. 2C). Exploratory times for the two objects did not differ across the groups (fig. S4, A and B). Consistent with cognitive function, the myelin density was significantly decreased in vehicle-administered BCAS-operated WT mice but not in cinnamaldehyde-administered BCAS-operated WT mice. The effect of cinnamaldehyde was significantly diminished in TRPA1-KO mice (Fig. 2, D and E). Together, TRPA1 stimulation ameliorates BCAS-induced outcomes, such as cognitive impairment and white matter injury.

### TRPA1 deficiency diminishes the BCAS-induced increase in astrocyte numbers

**Fig. 3.**
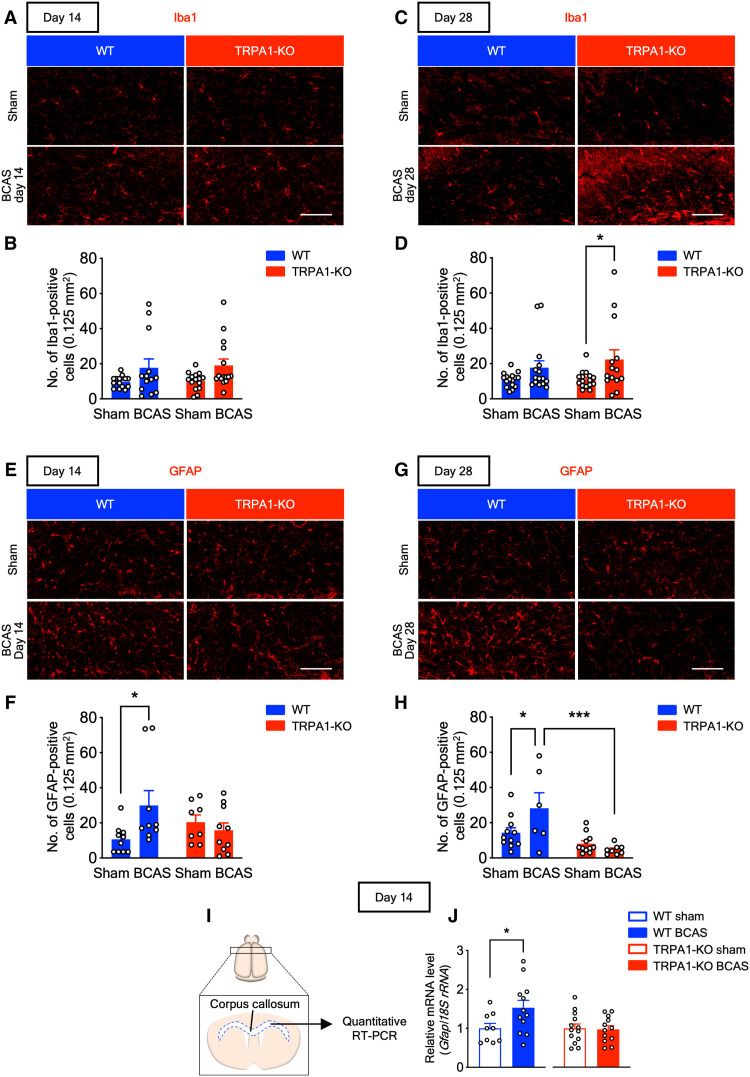
BCAS increases the number of GFAP-positive astrocytes in WT but not TRPA1-KO mice. (**A** to **D**) Representative images of immunostaining with anti-Iba1 antibody in the corpus callosum (A) and (C) and summarized data for the number of Iba1-positive cells (B) and (D) on days 14 (A) and (B) and 28 (C) and (D). (**E** to **H**) Representative images of immunostaining with anti-GFAP antibody in the corpus callosum (E) and (G) and summarized data for the number of GFAP-positive cells (F) and (H) on days 14 (E) and (F) and 28 (G) and (H). (**I**) A schematic of quantitative RT-PCR of the corpus callosum. (**J**) *Gfap* mRNA expression in the corpus callosum on day 14. Values are means ± SEM. Scale bars, 100 μm. (B) *n* = 13 to 15; (D) *n* = 14 to 16; (F) *n* = 8 to 10; (H) *n* = 6 to 11; (J) *n* = 9 to 12 (WT), *n* = 12 to 13 (TRPA1-KO). *P < 0.05 and ***P < 0.001 for two-way ANOVA with Bonferroni’s post hoc test (D), (F), and (H). *P < 0.05 for two-tailed unpaired Student’s *t* test (J).

Some studies suggest that glial activation is involved in the outcomes of VCI (*10*, *29*). To identify the cell types responsible for the differences in CCH-induced VCI and white matter damage between WT and TRPA1-KO mice, we performed immunostaining in the corpus callosum for Iba1 (a marker for microglia/macrophages; Fig. 3, A to D) and glial fibrillary acidic protein (GFAP; a marker for astrocytes; Fig. 3, E to H). The number of Iba1-positive cells tended to be increased in BCAS-operated mice, compared with sham-operated mice, with significant increases in TRPA1-KO mice on day 28 but not on day 14 (Fig. 3, A to D). By contrast, the number of GFAP-positive cells was increased in BCAS-operated WT mice, compared with sham-operated WT mice, but not in BCAS-operated TRPA1-KO mice on day 14 (Fig. 3, E and F). Similar to day 14, the number of GFAP-positive cells was increased in BCAS-operated WT mice, but not in BCAS-operated TRPA1-KO mice, on day 28 (Fig. 3, G and H). Next, we assessed *Gfap* mRNA expression in the corpus callosum with quantitative RT-PCR (Fig. 3I). *Gfap* mRNA expression was increased in BCAS-operated WT mice, but not in TRPA1-KO mice, on day 14 (Fig. 3J). These results suggest that TRPA1 deficiency suppresses the CCH-induced increase in GFAP-positive astrocyte numbers but does not affect Iba1-positive cell numbers.

### Astrocyte-specific TRPA1 deficiency accelerates BCAS-induced cognitive impairment and white matter injury

**Fig. 4.**
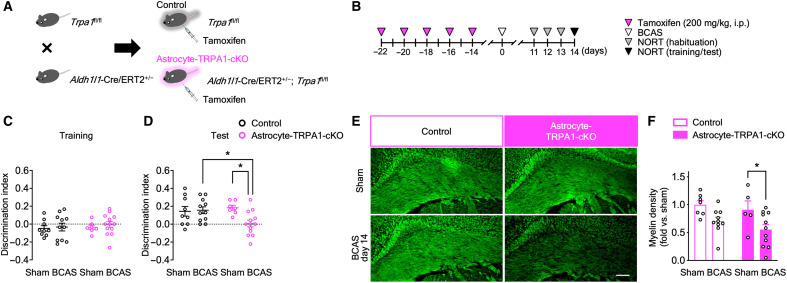
BCAS induces cognitive impairment and white matter injury on day 14 in astrocyte-TRPA1-cKO mice. (**A**) A schematic for obtaining tamoxifen-inducible astrocyte-TRPA1-cKO and control mice. (**B**) The experimental time course for tamoxifen administration and the NORT. (**C** and **D**) Discrimination indexes for exploring the blue quadrangular object during the training session (C) and the wooden ball, i.e., novel object, during the test session (D) on day 14. (**E** and **F**) Representative images of myelin staining in the corpus callosum (E) and summarized data for relative myelin density (F) on day 14. Values are means ± SEM. Scale bar, 100 μm. (C) and (D) *n* = 7 to 13; (F) *n* = 5 to 11. *P < 0.05 for two-way ANOVA with Bonferroni’s post hoc test (D) and (F).

Next, we determined the cells in which TRPA1 is functionally involved in VCI. TRPA1 is functionally expressed in brain cells, including astrocytes (*19*), endothelial cells (*22*), and oligodendrocytes (*23*). We generated conditional TRPA1-KO mice specific to astrocytes, endothelial cells, and oligodendrocyte lineage cells. For the generation of astrocyte-specific conditional TRPA1-KO (astrocyte-TRPA1-cKO) mice, we crossed homozygous *Trpa1*^fl/fl^ mice with hemizygous *Aldh1l1*-Cre/ERT2^+/−^ mice (Fig. 4A). To induce recombination, we intraperitoneally administered tamoxifen for five alternate days, and BCAS or sham operation was conducted 14 days after the last tamoxifen administration (Fig. 4B). During the NORT training session, on day 14, DI did not differ between the groups (Fig. 4C). During the test session, DI was significantly decreased in BCAS-operated astrocyte-TRPA1-cKO mice but not in BCAS-operated control mice (Fig. 4D). The exploratory times for the two objects did not differ, suggesting similar exploratory behavior across the groups (fig. S4, C and D). Consistent with cognitive function, myelin density was significantly decreased in BCAS-operated astrocyte-TRPA1-cKO mice but not in control mice on day 14 (Fig. 4, E and F).

In addition, we generated endothelial cell–specific conditional TRPA1-KO mice (endothelial cell–TRPA1-cKO) and oligodendrocyte lineage cell–specific conditional TRPA1-KO mice [OPC/oligodendrocyte (OL)–TRPA1-cKO] by crossing homozygous *Trpa1*^fl/fl^ mice with hemizygous *Tek*-Cre^+/−^ mice or *Pdgfra*-Cre^+/−^ mice (fig. S5, A, B, I, and J). Similar to astrocyte-TRPA1-cKO mice, on day 14, DI did not differ during the training session across the groups (fig. S5, C and K). In contrast to astrocyte-TRPA1-cKO mice, DI was not decreased in BCAS-operated endothelial cell–TRPA1-cKO or OPC/OL-TRPA1-cKO mice compared with the respective sham-operated mice during the test session (fig. S5, D and L). The exploratory times for the two objects were also the same across the groups (fig. S5, E, F, M, and N). Moreover, consistent with cognitive function, the myelin density was not decreased in BCAS-operated endothelial cell–TRPA1-cKO or OPC/OL-TRPA1-cKO mice on day 14 (fig. S5, G, H, O, and P). These results suggest that TRPA1 deficiency in astrocytes, but not in endothelial cells or oligodendrocyte lineage cells, accelerates CCH-induced VCI and white matter injury from early-stage CCH and that TRPA1 is functionally involved in astrocytes during early-stage CCH.

### BCAS up-regulates astrocyte-related genes in WT but not TRPA1-KO mice

**Fig. 5.**
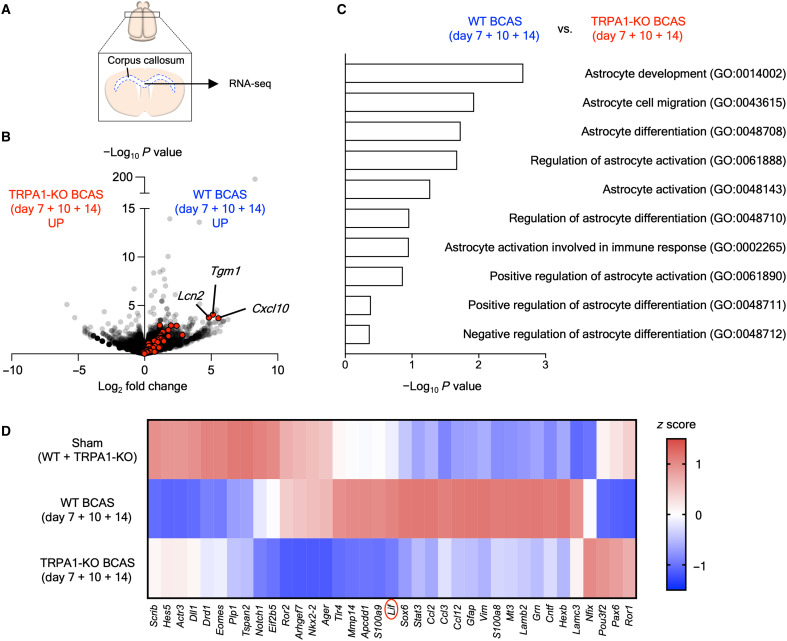
BCAS up-regulates astrocyte-related genes in WT but not TRPA1-KO mice. (**A**) A schematic of RNA-seq in the corpus callosum. (**B**) A volcano plot comparing all genes in the dataset between BCAS-operated WT and TRPA1-KO mice (average of days 7, 10, and 14). Red dots, reactive astrocyte marker genes. (**C**) The top 10 astrocyte-related terms in the GO enrichment analysis for differentially expressed genes between BCAS-operated WT and TRPA1-KO mice (average of days 7, 10, and 14). (**D**) A heatmap of the expression of genes contained in the top three astrocyte-related GO terms (GO:0014002, GO:0043615, and GO:0048708) in the sham-operated group (average of sham-operated WT and TRPA1-KO mice) and in BCAS-operated WT and TRPA1-KO mice (average of days 7, 10, and 14). *n* = 1.

To investigate the molecular mechanisms of TRPA1-mediated suppression of CCH-induced VCI and white matter injury, we conducted RNA sequencing (RNA-seq) analysis in the corpus callosum of sham- and BCAS-operated WT and TRPA1-KO mice (Fig. 5A). The number of GFAP-positive cells and level of *Gfap* mRNA expression in the corpus callosum were increased in BCAS-operated WT but not TRPA1-KO mice (Fig. 3), and it is well recognized that astrocytes exhibit a reactive profile in CNS injury and disease (*30*). Therefore, we investigated the expression of reactive astrocyte markers with RNA-seq. Reactive astrocyte marker genes (*30*) were up-regulated in BCAS-operated WT but not TRPA1-KO mice (Fig. 5B). In addition, Gene Ontology (GO) enrichment analysis for differentially expressed genes between BCAS-operated WT (days 7, 10, and 14) and TRPA1-KO mice (days 7, 10, and 14) revealed that astrocyte-related terms were enriched (Fig. 5C). To detect functional molecules through TRPA1 stimulation in astrocytes under CCH, we visualized the genes contained in the top three astrocyte-related GO terms (GO:0014002, GO:0043615, and GO:0048708) in a heatmap (Fig. 5D). The heatmap displayed up-regulation of 19 genes in BCAS-operated WT but not TRPA1-KO mice. Among them, LIF has been reported to promote myelination (*31*), protect against cuprizone-induced demyelination (*32*), improve neurological function in hypoxic-ischemic brain injury (*33*), and undergo release from astrocytes (*31*, *34*). Therefore, we hypothesized that LIF is a promising candidate that protects against VCI.

### TRPA1 deficiency diminishes the BCAS-induced increase of LIF

**Fig. 6.**
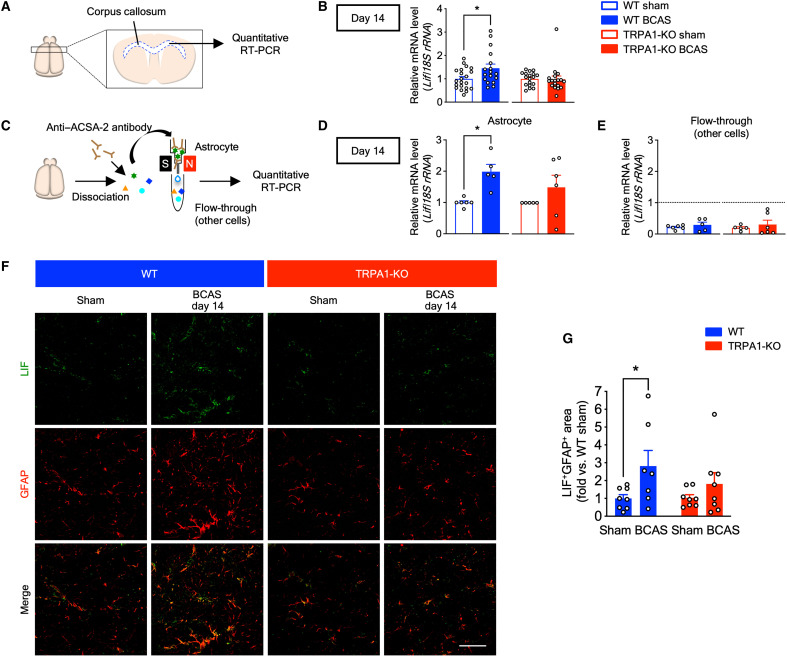
TRPA1 mediates LIF production in astrocytes in BCAS-operated WT mice on day 14. (**A**) A schematic of quantitative RT-PCR of the corpus callosum. (**B**) *Lif* mRNA expression in the corpus callosum. (**C**) A schematic of astrocyte isolation from the whole brain using MACS. (**D** and **E**) *Lif* mRNA expression in isolated astrocytes (D) and flow-through (E). (**F** and **G**) Representative images of immunostaining with anti-LIF antibody (green) and anti-GFAP antibody (red) in the corpus callosum (F), as well as summarized data for the percentage of LIF/GFAP double-positive surface areas (G) on day 14. Values are means ± SEM. Scale bar, 50 μm. (B) *n* = 17 to 21 (WT), *n* = 18 (TRPA1-KO); (D) and (E) *n* = 5 to 6 (WT), *n* = 5 to 6 (TRPA1-KO); (G) *n* = 7 to 8. *P < 0.05 for two-tailed unpaired Welch’s *t* test (B) and (D). *P < 0.05 for two-way ANOVA with Bonferroni’s post hoc test (G).

We next examined *Lif* mRNA expression in the corpus callosum on day 14 with quantitative RT-PCR (Fig. 6, A and B). *Lif* mRNA expression was increased in BCAS-operated WT mice compared with sham-operated mice, whereas there were no differences in TRPA1-KO mice (Fig. 6B). To assess *Lif* mRNA expression in astrocytes, we isolated astrocytes from whole adult mouse brain by magnetic-activated cell sorting (MACS) using the anti–astrocyte cell surface antigen-2 (ACSA-2) antibody (Fig. 6, C to E). *Lif* mRNA expression in isolated astrocytes was also increased in BCAS-operated WT but not TRPA1-KO mice (Fig. 6D). Conversely, *Lif* mRNA expression in the flow-through fraction, which is composed of cells other than astrocytes, was lower than in astrocytes and did not differ between sham- and BCAS-operated groups in both WT and TRPA1-KO mice (Fig. 6E). In addition, we performed immunohistochemistry in the corpus callosum and found that LIF was increased in GFAP-positive astrocytes in BCAS-operated WT but not TRPA1-KO mice (Fig. 6, F and G). To further investigate cellular sources of LIF, we performed double-fluorescent labeling immunohistochemistry using anti-LIF and anti-Iba1 antibody or anti-PDGFRα antibody in the corpus callosum. We found that LIF rarely merged with Iba1-positive microglia and PDGFRα-positive OPCs unlike GFAP-positive astrocytes (Fig. 6F and fig. S6). These results indicate that LIF are largely released from astrocytes in CCH-induced VCI.

Among the genes shown in Fig. 5D, ciliary neurotrophic factor (CNTF) is also known to promote myelination and is another protective candidate against VCI (*35*). Thus, we examined *Cntf* mRNA expression (fig. S7, A to C) and found that, similar to LIF, *Cntf* mRNA expression in the corpus callosum was increased in BCAS-operated WT mice compared with sham-operated mice, whereas there were no differences in TRPA1-KO mice (fig. S7A). In contrast to *Lif* mRNA expression, *Cntf* mRNA expression in isolated astrocytes was not increased in WT or TRPA1-KO mice. Furthermore, *Cntf* mRNA expression in the flow-through fraction also did not differ from that in astrocytes and did not differ between the groups (fig. S7, B and C). Moreover, immunostaining in the corpus callosum revealed no differences in the expression of CNTF in GFAP-positive astrocytes in WT and TRPA1-KO mice (fig. S7, D and E).

**Fig. 7.**
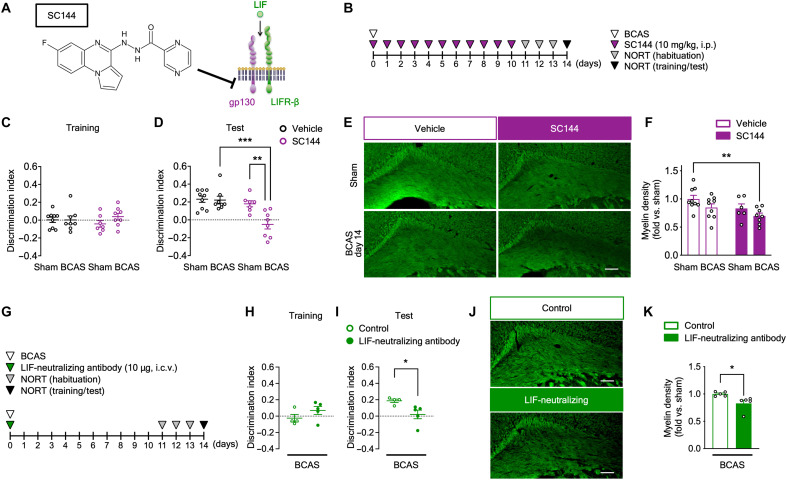
Inhibition of LIF signaling accelerates BCAS-induced cognitive impairment and white matter injury. (**A**) A schematic of the inhibitory mechanism of SC144. (**B** and **G**) The experimental time course for SC144 (B) or LIF-neutralizing antibody (G) administration and the NORT. (**C**, **D**, **H**, and **I**) Discrimination indexes for exploring the blue quadrangular object during the training session (C) and (H) and the wooden ball, i.e., novel object, during the test session (D) and (I) on postoperative day 14. (**E**, **F**, **J**, and **K**) Representative images of myelin staining in the corpus callosum (E) and (J) and summarized data for relative myelin density (F) and (K) on postoperative day 14. Values are means ± SEM. Scale bars, 100 μm. (C) and (D) *n* = 7 to 9; (F) *n* = 6 to 9; (H) and (I) *n* = 4 to 5; (K) *n* = 5. **P < 0.01 and ***P < 0.001 for two-way ANOVA with Bonferroni’s post hoc test (D). **P < 0.01 for two-way ANOVA with Dunnett’s post hoc test (F). *P < 0.05 for two-tailed unpaired Student’s *t* test (I). *P < 0.05 for two-tailed unpaired Welch’s *t* test (K). i.c.v., intracerebroventricular administration.

To further investigate the involvement of LIF in VCI, we used SC144, a selective inhibitor of gp130 [a component of LIF receptor complexes (*36*)] that reduces downstream signaling of gp130 ligands, such as LIF (*37*). We intraperitoneally administered SC144 on postoperative days 0 to 10 (Fig. 7, A and B). During the NORT training session, DI did not differ between the groups (Fig. 7C). Conversely, during the test session, DI was significantly decreased in BCAS-operated SC144-administered mice but not vehicle-administered mice (Fig. 7D). Exploratory times for the two objects were not different across the groups during the test session, but were decreased in SC144-administered BCAS-operated mice during the training session (fig. S7, F and G). Moreover, myelin density was also decreased in SC144-administered BCAS-operated mice (Fig. 7, E and F). We then intracerebroventricularly administered a LIF-neutralizing antibody once after BCAS operation (Fig. 7G). During the NORT training session, DI did not differ between the groups (Fig. 7H). Similar to the experiments with SC144, during the test session, DI was significantly decreased in LIF-neutralizing antibody-administered BCAS-operated mice compared with control antibody–administered mice (Fig. 7I). Exploratory times for the two objects were not different across the groups (fig. S7, H and I). Consistent with the results of cognitive impairment, myelin density was also decreased in LIF-neutralizing antibody-administered BCAS-operated mice compared with control antibody–administered mice (Fig. 7, J and K). These results suggest that TRPA1 in astrocytes is involved in the production of LIF during CCH and that increased LIF exerts positive effects on VCI-related outcomes.

### H_2_O_2_-elicited TRPA1-mediated astrocytic LIF promotes myelination of cultured OPCs

**Fig. 8.**
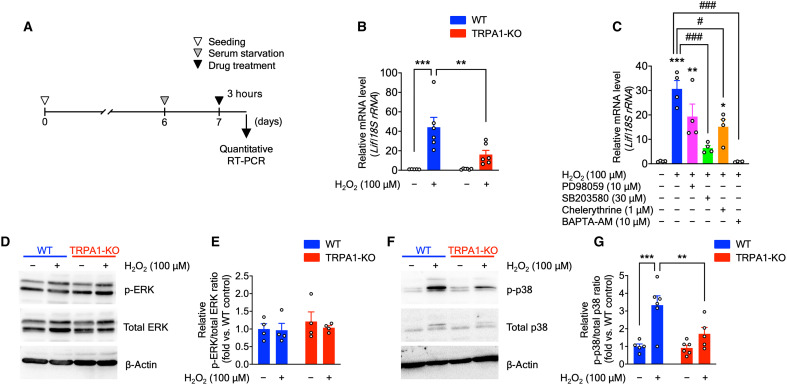
TRPA1 stimulation by H_2_O_2_ induces LIF production via p38-MAPK phosphorylation. (**A**) Experimental time course for in vitro experiments using primary astrocyte cultures. (**B**) *Lif* mRNA expression in primary astrocyte cultures treated with H_2_O_2_ (100 μM, 3 hours), determined by quantitative RT-PCR. (**C**) *Lif* mRNA expression in primary astrocyte cultures cotreated with H_2_O_2_ (100 μM) and ERK pathway inhibitor (PD98059; 10 μM), p38 inhibitor (SB203580; 30 μM), PKC inhibitor (chelerythrine; 1 μM), and Ca^2+^ signaling blocker (BAPTA-AM; 10 μM) for 3 hours, determined by quantitative RT-PCR. *P < 0.05, **P < 0.01, and ***P < 0.001 versus control; #*P* < 0.05 and ###*P* < 0.001 versus H_2_O_2_ for one-way ANOVA with Tukey’s post hoc test. (**D** to **G**) Representative immunoblot images of ERK (D) and p38 (F), as well as protein and phosphorylation levels of ERK (E) and p38 (G). Values are means ± SEM. (B) *n* = 5 to 6; (C) and (E) *n* = 4; (G) *n* = 5 to 6. **P < 0.01 and ***P < 0.001 for two-way ANOVA with Bonferroni’s post hoc test (B) and (G).

To further identify the molecular mechanisms underlying LIF production in astrocytes, we performed in vitro experiments using primary astrocyte cultures derived from WT or TRPA1-KO mice. We treated the cultures for 3 hours with H_2_O_2_, which stimulates TRPA1, and assessed *Lif* mRNA expression with quantitative RT-PCR (Fig. 8A). Expectedly, *Lif* mRNA expression was increased in H_2_O_2_-treated WT astrocytes but not TRPA1-KO astrocytes (Fig. 8B). Because a previous study demonstrated that protein kinase C (PKC), extracellular signal–regulated kinase 1/2 (ERK1/2), and p38–mitogen-activated protein kinase (MAPK) activation participates in LIF expression and release from astrocytes (*34*), we investigated the effects of these pathways on H_2_O_2_-induced *Lif* mRNA expression. We found that H_2_O_2_-induced *Lif* mRNA expression was significantly suppressed by the p38-MAPK inhibitor SB203580, partially suppressed by the PKC inhibitor chelerythrine, and not suppressed by the ERK pathway inhibitor PD98059 (Fig. 8C). Moreover, the intracellular Ca^2+^ chelator 1,2-bis-(o-aminophenoxy)-ethane-N,N,N',N'-tetraacetic acid, tetraacetoxymethyl ester (BAPTA-AM) significantly diminished H_2_O_2_-induced *Lif* mRNA expression (Fig. 8C). Consistent with these results, H_2_O_2_ induced p38-MAPK phosphorylation in WT but not TRPA1-KO astrocytes; however, ERK-MAPK phosphorylation was not observed by immunoblot analyses (Fig. 8, D to G). Together, H_2_O_2_-induced TRPA1 stimulation increases p38-MAPK phosphorylation and LIF production.

**Fig. 9.**
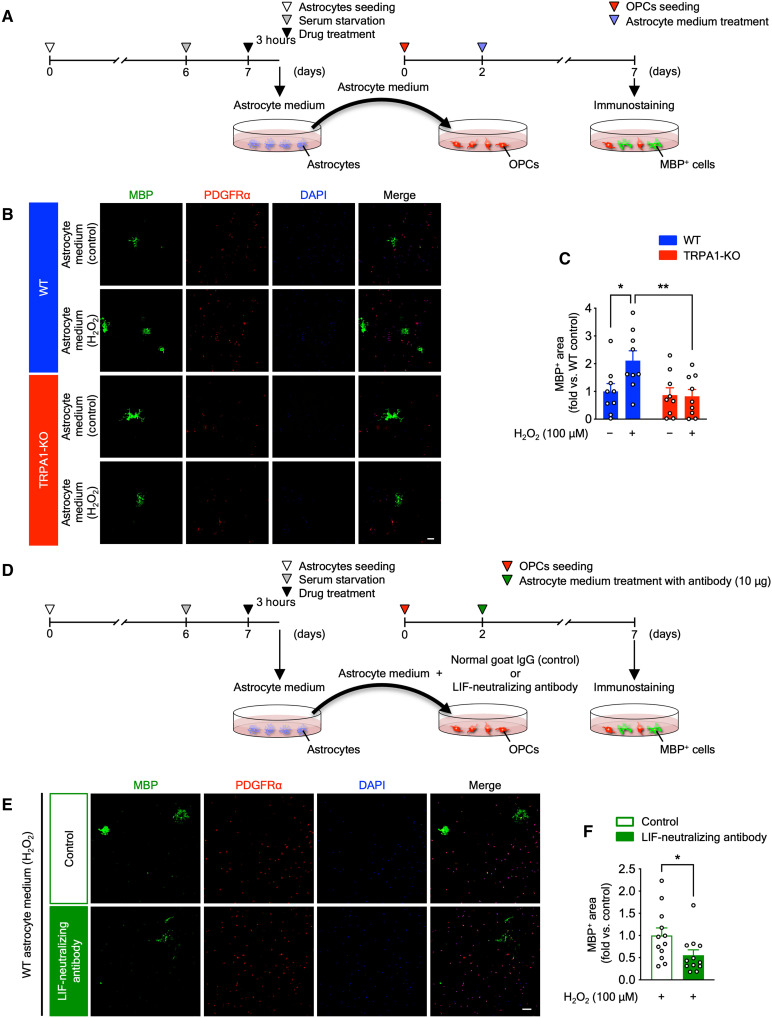
H_2_O_2_-elicited TRPA1-mediated astrocytic LIF promotes myelination of cultured OPCs. (**A**) Experimental time course for in vitro medium transfer experiments from astrocytes to OPCs. (**B** and **C**) Representative images of immunostaining with anti-MBP antibody (green), anti-PDGFRα antibody (red), and 4′,6-diamidino-2-phenylindole (DAPI) (blue) in primary OPC cultures treated with astrocyte medium (B) and summarized data for the percentage of MBP-positive surface areas (C). (**D**) Experimental time course for in vitro medium transfer experiments with a LIF-neutralizing antibody. (**E** and **F**) Representative images of immunostaining with anti-MBP antibody (green), anti-PDGFRα antibody (red), and DAPI (blue) in primary OPC cultures treated with H_2_O_2_-treated WT astrocyte medium with LIF-neutralizing antibody or control antibody (E) and summarized data for the percentage of MBP-positive surface areas (F). Values are means ± SEM. Scale bars, 100 μm. (C) *n* = 9; (F) *n* = 12. *P < 0.05 and **P < 0.01 for two-way ANOVA with Bonferroni’s post hoc test (C). *P < 0.05 for two-tailed unpaired Student’s *t* test (F).

Last, to investigate whether secretion factors, such as LIF, from H_2_O_2_-treated astrocytes promote OPC myelination, we performed in vitro medium transfer experiments. We collected culture medium from astrocytes (astrocyte medium) on day 7 in vitro, and OPC culture medium was switched from proliferation medium to astrocyte medium on day 2 in vitro (Fig. 9A). After incubation in astrocyte medium for 5 days, we performed immunostaining with anti-PDGFRα antibody (a marker for OPCs) and anti-MBP antibody (a marker for mature oligodendrocytes) to investigate OPC maturation. The area of MBP-positive mature oligodendrocytes was increased in H_2_O_2_-treated WT astrocyte medium compared with control, but not in H_2_O_2_-treated TRPA1-KO astrocyte medium (Fig. 9, B and C). To further investigate whether the effect of H_2_O_2_-treated WT astrocyte medium on OPC myelination is mediated by LIF, we also performed in vitro medium transfer experiments with LIF-neutralizing antibody. On day 2 in vitro, OPC culture medium was switched from proliferation medium to H_2_O_2_-treated WT astrocyte medium with LIF-neutralizing antibody or control antibody, and we performed immunostaining of PDGFRα and MBP after incubation in astrocyte medium for 5 days (Fig. 9D). The area of MBP-positive mature oligodendrocytes was significantly decreased in H_2_O_2_-treated WT astrocyte medium with LIF-neutralizing antibody compared with control antibody (Fig. 9, E and F). Together, astrocyte-derived LIF, which is produced by H_2_O_2_-induced TRPA1 stimulation, plays important roles in promoting OPC myelination.

## DISCUSSION

**Fig. 10.**
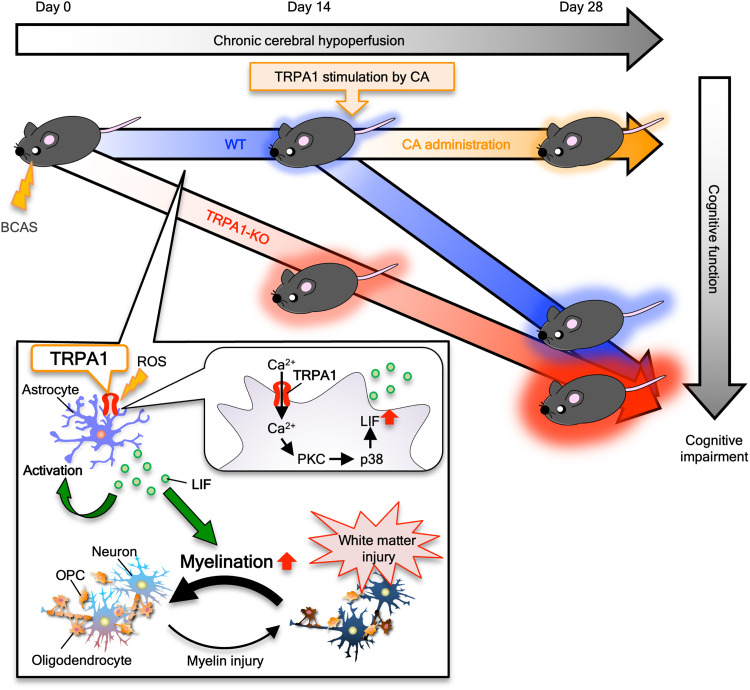
The astrocytic TRPA1 plays a protective role in CCH-induced VCI by producing LIF. A schematic summary of the role and mechanism of TRPA1 during CCH. TRPA1 stimulation in astrocytes under CCH increases LIF production by increasing Ca^2+^ influx and inducing p38-MAPK phosphorylation and promotes myelination. ROS, reactive oxygen species.

The findings of this study are as follows (Fig. 10): (i) TRPA1 deficiency accelerated BCAS-induced cognitive impairment and white matter injury during early-stage CCH (on day 14). (ii) TRPA1 stimulation with cinnamaldehyde on days 15 to 24 ameliorated BCAS-induced cognitive impairment and white matter injury. (iii) During early-stage CCH (on day 14), BCAS increased the number of GFAP-positive astrocytes in the corpus callosum, and TRPA1 deficiency diminished this increase in astrocytes. (iv) BCAS-induced cognitive impairment and white matter injury were accelerated by astrocyte-specific, but not by endothelial cell– or oligodendrocyte lineage cell–specific, TRPA1 deficiency. (v) TRPA1 deficiency diminished the BCAS-induced increase of LIF expression in astrocytes during early-stage CCH (on day 14). (vi) In primary astrocyte cultures, LIF expression was increased in H_2_O_2_-treated WT but not in TRPA1-KO astrocytes, and culture medium from H_2_O_2_-treated WT astrocytes promoted OPC myelination. Together, these results indicate that, through LIF production, TRPA1 in astrocytes plays protective roles in preventing the onset of early CCH pathogenesis.

Intra-brain vascular dysregulation has been reported to occur earlier than other pathologic events including amyloid-β deposition and hyperphosphorylation of tau during AD development (*38*), and more than 80% of aged participants show morphological substrates of cerebrovascular disease in addition to AD pathology (*7*), indicating the importance of SVD in both AD and vascular dementia. Recent studies have strongly suggested that CCH may be a major factor in SVD (*7*, *8*). Deschaintre *et al.* (*39*) reported that treatment of lifestyle-related diseases, which elicit CCH-associated SVD, such as diabetes, dyslipidemia, and hypertension, improves dementia. Moreover, catheter ablation therapy for patients with atrial fibrillation, who are at risk of dementia (*40*), improves cognitive function by restoring cardiac function and by mitigating CCH (*41*), suggesting that ameliorating CCH is also a promising approach for overcoming VCI.

It would be of interest to demonstrate the protective role of TRPA1 during the early pathogenesis of VCI, and to which stimuli TRPA1 opens. The most likely candidate molecules for TRPA1 stimulation are reactive oxygen species (ROS), including H_2_O_2_. TRPA1 is the most redox-sensitive TRP channel (*13*), is more sensitized and likely to open during hypoxia (*42*, *43*), and is activated by H_2_O_2_ (*44*). Actually, H_2_O_2_-induced Ca^2+^ influx through TRPA1, but not TRPM2, induces oxidative stress tolerance in cancer cells (*45*). In addition, H_2_O_2_ enhances TRPA1-dependent as well as TRPV1- and TRPM3-independent heat responses in sensory neurons (*46*). In our CCH-induced VCI model, H_2_O_2_ levels are significantly elevated 14 days after BCAS compared with the sham-operated group (*9*). ROS are assumed to play a paradoxical role in the pathogenesis of VCI during early-stage CCH, compared with middle- and late-stage CCH. It has been reported that ROS are produced and exert detrimental effects during the late stages of BCAS-induced VCI (*9*, *47*). In this context, we have previously reported that TRPM2, which is a ROS-sensitive TRP channel and functionally expressed in microglia, aggravates CNS inflammation, white matter injury, and cognitive impairment in the middle to late stages of BCAS-induced VCI (*9*). Conversely, it has also been reported that ROS are not always cytotoxic; instead, moderate ROS levels exert positive effects on cell homeostasis (*48*). Mild oxidative stress activates the astrocytic antioxidant response and contributes to neuronal survival in ischemic preconditioning (*49*). In our study, on postoperative day 14, cognitive dysfunction was observed in TRPA1-KO but not WT mice. Therefore, TRPA1, a ROS-sensitive channel, may play a preventive role during the early-stage CCH.

Furthermore, it is important in which brain cells TRPA1 participate in early-stage CCH pathogenesis. In the present study, in vitro experiments, immunostaining, and quantitative RT-PCR of brain sections demonstrated that TRPA1 mediates astrocytic functions. In addition, we generated cell-specific TRPA1-deficient mice using the Cre recombinase–loxP system. With this, we showed that only astrocyte-specific, but not endothelial cell– or oligodendrocyte lineage cell–specific, TRPA1-deficient mice exhibited aggravated cognitive impairment and white matter injury during early-stage CCH. TRPA1 is functionally expressed in astrocytes and regulates astrocytic Ca^2+^ levels (*19*–*21*). Moreover, TRPA1 in astrocytes mediates the selective sensing of moderate hypoxia in the parafacial respiratory group and retrotrapezoid nucleus regions (*50*). TRPA1 is also reported to be present in cerebral artery endothelial cells and to mediate cerebral artery dilation (*22*, *51*). Furthermore, TRPA1 in cerebral endothelial cells is a sensor of hypoxia, and acute hypoxic exposure increased TRPA1 sparklet frequency, leading to vasodilation; hence, TRPA1 plays a protective role in cerebral ischemic stroke (*24*). Conversely, TRPA1 is also expressed in oligodendrocytes, and TRPA1 blockade reduces myelin damage in mimicking ischemia (*23*).

It is unclear why TRPA1 in oligodendrocytes and cerebral artery endothelial cells was not involved in VCI in this study. The CCH-induced VCI model used in this study is a mild pathological model that does not induce neuronal death or mouse paralysis in vivo, outcomes that are, however, induced by the ischemic brain injury model involving TRPA1 in oligodendrocytes (*23*) and cerebral artery endothelial cells (*24*). Therefore, the cells in which TRPA1 responds to stimuli may depend on different pathologies. TRPA1 in cerebral artery endothelial cells or oligodendrocytes may play a critical role under severe hypoxic conditions (e.g., ischemic stroke). By contrast, TRPA1 in astrocytes may play a critical role in environments in which small amounts of ROS are produced (e.g., BCAS-induced VCI) and in which the innate defense system can protect against CNS damage. Nevertheless, further investigations regarding the involvement of TRPA1 in endothelial cells and oligodendrocytes are required.

Emerging evidence indicates that LIF plays vital roles in the CNS (*52*). LIF is released from astrocytes, promotes myelination (*31*), protects against cuprizone-induced demyelination (*32*), and improves myelination and neurological function in hypoxic-ischemic brain injury (*33*). We have shown that BCAS-induced VCI increased LIF expression in WT but not TRPA1-KO astrocytes on day 14 (i.e., early-stage CCH). This indicates that signaling through TRPA1 openings suppresses the early pathology associated with VCI. Moreover, in this study, H_2_O_2_ treatment increased *Lif* mRNA expression in WT astrocytes, and H_2_O_2_-treated WT astrocyte medium induced myelination of primary OPCs. This suggests that astrocyte-derived LIF may promote remyelination as a compensatory mechanism against white matter injury in VCI. In addition, LIF induces the differentiation (*53*) and responses of astrocytes to CNS injury (*54*). Selective deletion of signal transducer and activator of transcription 3 (STAT3), which is activated by various cytokines such as LIF, from astrocyte markedly attenuates various aspects of reactive astrogliosis, including cell hypertrophy, up-regulation of GFAP, and scar formation after spinal cord injury (*55*–*57*). Actually, we performed immunostaining of GFAP in the experiments with SC144 and observed that the number of GFAP-positive cells was lower in SC144-administered BCAS-operated mice than vehicle-administered BCAS-operated mice (fig. S8, A and B). In damaged CNS tissues, astrocytes contribute to neuroprotection, maintenance of the blood-brain barrier (BBB), and regulation of inflammation (*58*, *59*). These imply that LIF released from astrocytes acts not only on OPCs to promote myelination but also on neighboring astrocytes via STAT3-dependent mechanism and may generally contribute to repairing injured CNS tissue.

Therefore, we compared the BBB function of WT and TRPA1-KO mice on day 14 (i.e., during early-stage CCH). WT mice exhibited significantly increased astrocyte numbers and no significant changes in the permeability of the BBB to sodium fluorescein. Conversely, TRPA1-KO mice exhibited unchanged astrocyte numbers and significantly increased the permeability of the BBB to sodium fluorescein (Fig. 3 and fig. S8, C and D), indicating that TRPA1 deficiency induces astrocyte dysfunction, resulting in a failure to maintain BBB integrity during early-stage CCH. This further implies that LIF is released as an autocrine and/or paracrine extracellular signal to coerce astrocytes into contributing to the maintenance of neurovascular unit integrity and that astrocyte-derived LIF also protects against BBB damage in VCI. However, further studies on other protective mechanisms of LIF in VCI, such as inflammatory responses in the CNS, are needed.

Regarding signaling downstream of TRPA1 opening, previous studies have shown that TRPA1 participates in the up-regulation of interleukin-6 (IL-6) family cytokines (including LIF) in chondrocytes (*60*) and that adenosine receptor–mediated LIF expression and release from astrocytes require PKC (but not PKA) activation and depend on ERK1/2- and p38- (but not Janus kinase–) MAPK activation (*34*). In this study, the p38 MAPK inhibitor SB203580 and Ca^2+^ signaling blocker BAPTA-AM, but not ERK pathway inhibitor PD98059, suppressed the H_2_O_2_-induced increase in *Lif* mRNA expression. In addition, p38-MAPK phosphorylation was observed in H_2_O_2_-treated WT but not TRPA1-KO astrocytes, whereas there was no difference in ERK-MAPK phosphorylation. Together, H_2_O_2_ stimulates TRPA1, leading to increased Ca^2+^ influx, p38-MAPK phosphorylation, and LIF production in astrocytes, resulting in enhanced OPC myelination. However, further studies are needed to clarify the involvement of other pathways and molecules in the production of LIF in astrocytes.

In conclusion, TRPA1 plays a protective role in CCH-induced cognitive impairment and white matter injury by activating astrocytes, increasing LIF production, and promoting OPC myelination. A series of astrocytic TRPA1-LIF and myelination axis in VCI is the intrinsic protective mechanism. Together, the present study suggests that TRPA1 in astrocytes may be a promising therapeutic target for VCI.

## MATERIALS AND METHODS

### Animals

All animal experiments were conducted in accordance with the ethical guidelines of the Kyoto University animal experimentation committee and the Japanese Pharmacological Society. All animal use and study protocols were approved by the Kyoto University animal experimentation committee (approval number: 20-42). Male C57BL/6J WT mice (RRID: IMSR_JAX:000664) were purchased from Japan SLC, and TRPA1-KO mice were obtained from the Jackson Laboratory (Bar Harbor, ME; #006401). Mice were maintained in our laboratory and used at 8 to 12 weeks old and 20 to 30 g. TRPA1-KO mice were backcrossed to C57BL/6J mice for at least 10 generations and genotyped by genomic PCR using the following primers: 5′-tca tct ggg caa caa tgt cac ctg ct-3′ and 5′-tcc tgc aag ggt gat tgc gtt gtc ta-3′. All mice were housed at a constant ambient temperature of 22° ± 2°C under a 12-hour light/12-hour dark cycle and were allowed water ad libitum. The numbers of used animals were empirically determined and were similar to previous reports (*9*, *29*, *47*).

### Bilateral common carotid artery stenosis

Mice were subjected to BCAS using microcoils with an internal diameter of 0.18 mm (Sawane Spring, Shizuoka, Japan), as previously described (*9*, *29*). First, mice were anesthetized with 3% isoflurane in 30% O_2_ and 70% N_2_O and maintained on 1.5% isoflurane in 30% O_2_ and 70% N_2_O using a face mask. After a midline skin incision, the bilateral common carotid artery was isolated, and a microcoil was applied to it. Sham operation was performed in the same fashion as the BCAS operation, but without using a microcoil. The common carotid artery was isolated, while the sham control mice were subjected to anesthesia for the same amount of time as the BCAS-operated mice. Regional cerebral blood flow in the middle cerebral artery territory was monitored by laser Doppler flowmetry (Omegaflow, Omegawave). A flexible probe was fixed to the skull (2 mm posterior and 6 mm lateral to the bregma) before BCAS, and regional cerebral blood flow was monitored 60 min after operation. Mice were randomly assigned to either the BCAS or sham group.

### Generation of astrocyte-TRPA1-cKO

For the generation of astrocyte-TRPA1-cKO mice, we crossed mice homozygous for the loxP-flanked TRPA1 S5/S6 transmembrane domain construct (“*Trpa1*^fl/fl^”; Jackson Laboratory, #008650) with mice hemizygous for Cre recombinase under the control of the *Aldh1l1* promoter/enhancer (“*Aldh1l1*-Cre/ERT2^+/−^”; Jackson Laboratory, #029655). Cre-positive F_1_ progenies were crossed with homozygous *Trpa1*^fl/fl^ mice. F_2_ progenies were genotyped by PCR with genomic DNA obtained from tail biopsies, and those positive for Cre and homozygous for floxed TRPA1 were crossed with homozygous *Trpa1*^fl/fl^ mice. Astrocyte-TRPA1-cKO (*Aldh1l1*-Cre/ERT2^+/−^; *Trpa1*^fl/fl^) mice were viable and fertile. Control mice were homozygous for floxed TRPA1 but negative for Cre (*Trpa1*^fl/fl^). To induce recombination, all mice (both astrocyte-TRPA1-cKO and control mice) used in the experiments were intraperitoneally administered tamoxifen (200 mg/kg in corn oil, once per day) for five alternate days, using a previously reported method with some modifications (*61*). BCAS or sham operation was performed 14 days after the last tamoxifen administration. At the first tamoxifen administration, all mice were 6 to 9 weeks old and weighed 20 to 30 g. The BCAS or sham operation was conducted on 9- to 12-week-old mice.

### Generation of endothelial cell–TRPA1-cKO

For the generation of endothelial cell–TRPA1-cKO mice, we crossed homozygous *Trpa1*^fl/fl^ mice with mice hemizygous for Cre recombinase under the control of the *Tek* promoter/enhancer (“*Tek*-Cre^+/−^”) (RBRC04495) (*62*); the mouse strain was provided by RIKEN BRC through the National BioResource Project of MEXT/AMED, Japan. Cre-positive F_1_ progenies were mated with homozygous *Trpa1*^fl/fl^ mice. F_2_ progenies were genotyped by PCR with genomic DNA obtained from tail biopsies, and those positive for Cre and homozygous for floxed TRPA1 were crossed with homozygous *Trpa1*^fl/fl^ mice. Endothelial cell–TRPA1-cKO (*Tek*-Cre^+/−^; *Trpa1*^fl/fl^) mice were viable and fertile. Control mice were homozygous for floxed TRPA1 but negative for Cre (*Trpa1*^fl/fl^). The BCAS or sham operation was conducted on 8- to 12-week-old mice (25 to 35 g).

### Generation of OPC/OL-TRPA1-cKO

For the generation of OPC/OL-TRPA1-cKO mice, we crossed homozygous *Trpa1*^fl/fl^ mice with mice hemizygous for Cre recombinase under the control of the *Pdgfra* promoter/enhancer (“*Pdgfra*-Cre^+/−^”; Jackson Laboratory, #013148). Cre-positive F_1_ progenies were crossed with homozygous *Trpa1*^fl/fl^ mice. F_2_ progenies were genotyped by PCR with genomic DNA obtained from tail biopsies, and those positive for Cre and homozygous for floxed TRPA1 were crossed with homozygous *Trpa1*^fl/fl^ mice. OPC/OL-TRPA1-cKO (*Pdgfra*-Cre^+/−^; *Trpa1*^fl/fl^) mice were viable and fertile. Control mice were homozygous for floxed TRPA1 but negative for Cre (*Trpa1*^fl/fl^). The BCAS or sham operation was conducted on 8- to 12-week-old mice (20 to 30 g).

### Drug administration

Mice were intraperitoneally injected with trans-cinnamaldehyde (100 mg/kg; Tokyo Chemical Industry, Tokyo, Japan) once per day on postoperative days 15 to 24. Control groups were administered with vehicle solution [0.5% Tween 80 (Nacalai Tesque, Kyoto, Japan) in saline]. In addition, mice were intraperitoneally injected with SC144 (10 mg/kg; MedChemExpress) once per day on postoperative days 0 to 10. Control groups were administered with vehicle (10% dimethyl sulfoxide in 40% propylene glycol in saline).

### Neutralizing antibody administration

Mice were intracerebroventricularly injected with a goat polyclonal LIF-neutralizing antibody [10 μg; 1 mg/ml in phosphate-buffered saline (PBS); R&D Systems, catalog no. AB-449-NA] immediately after BCAS operation. Control groups were injected with a normal goat immunoglobulin G (IgG; 10 μg; 1 mg/ml in PBS; R&D Systems, catalog no. AB-108-C).

### Primary cultures of mouse astrocytes

Primary astrocyte-enriched cultures were prepared from the cerebral cortices of 0- to 2-day-old postnatal WT or TRPA1-KO C57BL/6J mice, as previously reported (*63*). Cells were dissociated and plated in 75-cm^2^ flasks with 7 ml of Eagle’s minimum essential medium (EMEM; Nissui Pharmaceutical, Tokyo, Japan) supplemented with 10% heat-inactivated fetal bovine serum (FBS; JRH Biosciences, Lenexa, KS, USA) in an incubator with 5% CO_2_ at 37°C. After cultivation for 2 to 4 weeks, the flasks were shaken at 400 rpm for 10 min using an orbital shaker to remove microglia. The supernatant was removed, the fresh culture medium was added, and the cultures were incubated in an incubator with 5% CO_2_ at 37°C for 2 hours. To remove OPCs, the flasks were then shaken on an orbital shaker at 250 rpm for 16 to 18 hours at 37°C. The remaining astrocytes were detached with 0.25% trypsin (Nacalai Tesque), resuspended in fresh medium, and reseeded on 35 mm dishes (20 × 10^4^ cells) or 60 mm dishes (40 × 10^4^ cells). After incubation with serum-free EMEM for 16 to 18 hours, cells were treated with drugs on day 7 after replating. Cells were used for experiments 3 hours after drug treatment. In addition, we used the following reagents: H_2_O_2_ (100 μM; Nacalai Tesque), PD98059 (10 μM; Cayman), SB203580 (30 μM; Cayman), chelerythrine chloride (1 μM; AdipoGen Life Sciences), and BAPTA-AM (10 μM; Nacalai Tesque).

### Primary cultures of mouse OPCs

Primary OPC-enriched cultures were prepared from the cerebral cortices of 0- to 2-day-old postnatal WT C57BL/6J mice. After isolating the cerebral cortices, cells were incubated with trypsin (2.5 mg/ml; Nacalai Tesque) and deoxyribonuclease I (DNase I; 10 mg/ml; Sigma-Aldrich) for 15 min at 37°C. Dissociated cells were plated onto poly-l-ornithine–coated 75 cm^2^ flasks in Advanced DMEM/F12 medium (Dulbecco’s modified Eagle’s medium/Ham’s F-12, Invitrogen) containing 10% heat-inactivated FBS and 1% penicillin/streptomycin mixed solution (Nacalai Tesque) and were maintained in an incubator with 5% CO_2_ at 37°C. After 10 to 12 days, OPCs were purified from the mixed glial culture via a two-step procedure. First, microglia were removed by shaking at 160 rpm for 2 hours using an orbital shaker. After removing the supernatant and adding fresh culture medium, OPCs on a monolayer of astrocytes were detached by shaking at 230 rpm for 16 to 18 hours. The supernatant was cultured on noncoated 10 cm dishes for 1.5 hours at 37°C in 5% CO_2_ to remove astrocytes. The final cell suspension was replated onto poly-l-ornithine–coated 10 mm glass coverslips (0.6 × 10^4^ cells) and maintained in Neurobasal medium (Invitrogen) supplemented with 1 × B27 (Invitrogen), glutamine (2 mM; Nacalai Tesque), 1% penicillin/streptomycin mixed solution, PDGF-AA (10 ng/ml; PeproTech), and basic fibroblast growth factor (10 ng/ml; PeproTech); this was used as a proliferation medium. Cells were used for medium transfer experiments 2 days after replating.

### Preparation of medium for astrocyte cultures

For medium transfer experiments, culture medium for astrocytes was changed to Neurobasal medium supplemented with 1 × B27, glutamine (2 mM), and 1% penicillin/streptomycin mixed solution 6 days after replating astrocytes. This was followed by incubation for 16 to 18 hours. After treatment with or without H_2_O_2_ (100 μM), the culture medium was collected, centrifuged at 2000*g* for 5 min at 4°C to remove cells and debris, and stored at −80°C until use, similar to a previous report (*64*).

### Novel object recognition test

Cognitive assessment by NORT was performed on postoperative days 14 and 28 as previously reported (*9*, *15*, *29*). Experiments were conducted under dim illumination (30 lux), with mice being habituated to the black box (30 cm by 30 cm by 30 cm) for 3 days (10 min/day) before the training. In the training session, two different objects (a yellow triangular prism and a blue quadrangular pyramid) were placed in the box, and mice were allowed to freely interact with the objects for 10 min. After 6 hours, the test session was performed, and the blue quadrangular object was replaced by a wooden ball that served as a novel object. The total exploratory time was defined as the time spent exploring both objects and was considered an indicator of locomotor activity. The DI was calculated as (the time spent exploring the blue quadrangular object − the time spent exploring the yellow triangular prism)/(the time spent exploring the blue quadrangular object + the time spent exploring the yellow triangular prism) in the training session or (the time spent exploring the wooden ball object − the time spent exploring the yellow triangular prism)/(the time spent exploring the wooden ball object + the time spent exploring the yellow triangular prism). This index was considered an indicator of recognition memory. Mice that spent <8 s exploring both objects were excluded.

### Novel location recognition test

The test apparatus and habituation/training procedure were identical to those described for NORT, as previously reported (*15*). During the test session, the blue quadrangular object was moved to a different location. As mentioned above, the total exploratory time was defined as the time spent exploring both objects and was considered an indicator of locomotor activity. DI was calculated as (the time spent exploring the blue quadrangular object − the time spent exploring the yellow triangular prism)/(the time spent exploring the blue quadrangular object + the time spent exploring the yellow triangular prism) and was considered an indicator of spatial memory.

### Myelin staining

Mice were intraperitoneally injected with pentobarbital (50 mg/kg of body weight) or a cocktail of three different anesthetic agents [medetomidine (0.3 mg/kg), midazolam (4.0 mg/kg), and butorphanol (5.0 mg/kg)] and were perfused transcardially with 0.1 M K^+^-free PBS followed by 4% paraformaldehyde in 0.1 M phosphate buffer (PB), using a previously reported method with some modifications (*65*). Brains were stored in paraformaldehyde for 3 hours and then transferred to 15% sucrose in 0.1 M PB at 4°C overnight. Coronal sections (20 μm thick) were cut using a cryostat (Leica Biosystems). To stain myelin, brain sections were soaked in 0.1% Triton X-100 in PBS for 20 min. The sections were incubated with the FluoroMyelin Green Fluorescent Myelin Stain (1:1000; Invitrogen, catalog no. F34651) for 20 min at room temperature. Fluorescence was visualized with the FluoView FV10i confocal microscope (Olympus) equipped with a laser scanning confocal imaging system. The mean intensity of FluoroMyelin staining in the corpus callosum was measured in a 200 μm by 200 μm field at approximately the bregma +0.7 mm.

### Transmission electron microscopy

TEM was performed as previously described (*16*). Mice were intraperitoneally injected with a cocktail of three different anesthetic agents [medetomidine (0.3 mg/kg), midazolam (4.0 mg/kg), and butorphanol (5.0 mg/kg)] and were perfused transcardially with 0.1 M K^+^-free PBS followed by 4% paraformaldehyde/2% glutaraldehyde in 0.1 M PB. The corpus callosum was then removed and dissected. After postfixing overnight at 4°C in 4% paraformaldehyde/2% glutaraldehyde, the corpus callosum was exposed to 1% osmium tetroxide in 0.1 M PB (pH 7.4) for 2 hours at room temperature, dehydrated by immersion in a series of graded ethanol solutions, and embedded in epoxy resin (Luveak 812, Nacalai Tesque) according to the inverted beam capsule procedure. Samples were polymerized at 60°C for 3 days. Ultrathin sections were prepared on an ultramicrotome (EM UC6, Leica) and stained with uranyl acetate and lead citrate. Last, sections were observed under a Hitachi H-7650 transmission electron microscope. Images obtained from each mouse were analyzed about demyelinated fibers and myelin thickness in a visual way. Fibers that have loosely wrapped myelin and cavities between myelin lamellae were regarded as “demyelinated” and counted manually. Myelin thickness was manually measured in the most inner layer using ImageJ software.

### Immunohistochemistry

#### Fluorescence staining

The coronal sections were blocked with PBS containing 3% bovine serum albumin and 0.1% Triton X-100 for 40 to 60 min. Next, they were incubated at 4°C overnight with the following primary antibodies: rabbit anti-Iba1 (1:500; Wako Pure Chemical, catalog no. 019-19741), rabbit anti-GFAP (1:5000; Abcam, catalog no. ab7260), mouse anti-APC (CC1) (1:200; Calbiochem, catalog no. OP80), rat anti-LIF (1:500; Novus Biologicals, catalog no. NBP2-27406), goat anti-CNTF (1:100; R&D Systems, catalog no. AF-557-NA), mouse anti-MBP (1:500; BioLegend, catalog no. 836504), rabbit anti-PDGFRα (1:1000; Santa Cruz Biotechnology, catalog no. sc-338), rabbit anti-OLIG2 (1:1000; GeneTex, catalog no. GTX132732), or rabbit anti-SOX10 (1:100; Abcam, catalog no. ab155279). The sections were then labeled at room temperature for 1.5 hours in the dark with the following secondary antibodies: Alexa Fluor donkey anti-rabbit (488, catalog no. A21206; 594, catalog no. A21207; or 647, catalog no. A31573), anti-mouse (488, catalog no. A21202), anti-rat (488, catalog no. A21208), or anti-goat (594, catalog no. A11058) IgG (1:300; Invitrogen). Fluorescence images were acquired using the FluoView FV10i confocal microscope (Olympus). The numbers or the areas of the following cells in the corpus callosum at the bregma +0.7 mm were counted: Iba1-, GFAP-, and CC1-positive cells (in a 0.125 mm^2^ field), as well as LIF/GFAP double-positive and CNTF/GFAP double-positive cells (in a 0.0450 mm^2^ field).

#### NeuN staining

Coronal sections were blocked with PBS containing 3% bovine serum albumin and 0.1% Triton X-100 for 60 min and then incubated with the primary mouse anti-NeuN antibody (1:500; Chemicon, catalog no. MAB377B) at 4°C overnight. Sections were then labeled with the biotinylated secondary antibody horse antimouse IgG (1:200; Vector Laboratories, catalog no. BA-2000) for 2 hours, followed by ABC Elite reagent (1:200; Vector Laboratories) for 1.5 hours. Immunoreactivity was visualized using diaminobenzidine (Nacalai Tesque) as a chromogen. The number of NeuN-positive cells in the hippocampus at the bregma −2.8 mm was evaluated.

### Immunocytochemistry

OPCs were cultured on 10 mm glass coverslips for 2 days. To analyze differentiation, cells were cultured for an additional 5 days in transferred astrocyte medium with or without LIF-neutralizing antibody (10 μg; R&D Systems, catalog no. AB-449-NA) or normal goat IgG (10 μg; R&D Systems, catalog no. AB-108-C). Cells were fixed in 4% paraformaldehyde and blocked with PBS containing 3% bovine serum albumin and 0.1% Triton X-100 for 10 min. Cells were incubated at 4°C overnight with primary antibodies against PDGFRα (rabbit anti-PDGFRα, 1:1000; Santa Cruz Biotechnology, catalog no. sc-338) and MBP (mouse anti-MBP, 1:500; BioLegend, catalog no. 836504). Cells were then labeled with the secondary antibodies Alexa Fluor donkey anti-rabbit (594) and anti-mouse (488) IgG (1:300; Invitrogen) at room temperature for 1.5 hours in the dark. After staining nuclei with 4′,6-diamidino-2-phenylindole (DAPI) Fluoromount-G (Southern Biotechnology Associates), fluorescence images were acquired using the FluoView FV10i confocal microscope (Olympus).

### Western blotting

Immunoblotting analyses were conducted on whole-cell lysates, as previously described (*66*). Primary astrocyte cultures grown in 60 mm dishes were harvested and lysed in radioimmunoprecipitation assay buffer with SDS (Nacalai Tesque) supplemented with 0.1% phosphatase inhibitor cocktails 2 and 3 (Sigma-Aldrich). Aliquots of lysate were diluted with an equal volume of sample buffer [50 mM tris-HCl, 10% glycerol, 2% SDS, and 6% 2-mercaptoethanol with bromophenol blue (pH 6.8)] and loaded onto a 10% SDS-polyacrylamide gel. Proteins were blotted onto Immobilon-P polyvinylidene difluoride transfer membranes (Millipore). The membranes were exposed to a blocking step and then incubated overnight at 4°C with the following primary antibodies: rabbit anti–phospho-p38-MAPK (1:500; catalog no. 4511), rabbit anti-p38 MAPK (1:500; catalog no. 9212), rabbit anti–phospho-p44/42 MAPK (Erk1/2) (1:1000; catalog no. 4370), rabbit anti-p44/42 MAPK (Erk1/2) (1:1000; catalog no. 9102; all from Cell Signaling Technology), or mouse anti–β-actin (1:20,000; Sigma-Aldrich, catalog no. A1978). The next day, the membranes were briefly washed with tris-buffered saline containing 0.1% (v/v) Tween 20 and then incubated for 1 hour at room temperature with the peroxidase-conjugated secondary antibodies donkey anti-rabbit IgG (1:5000; Cytiva, catalog no. NA934V) and goat anti-mouse IgG (1:20,000; Jackson ImmunoResearch, catalog no. 115-035-003). Specific bands were then detected with Immobilon Western chemiluminescent horseradish peroxidase substrate (Millipore).

### RNA sequencing

Total RNA was isolated from the corpus callosum using ISOGEN reagent (Nippon Gene) according to the manufacturer’s protocols. For RNA-seq analysis, polyadenylated RNA was selected from total RNA and reverse transcribed. A library was synthesized using the NEBNext Ultra RNA Library Prep Kit for Illumina, according to the manufacturer’s instructions. The prepared library was sequenced using NovaSeq 6000. The clean reads were mapped to the mouse reference genome GRCm38 using HISAT version 2.1.0 (*67*). Gene expression was calculated using subread version 2.0.1 (*68*) and edgeR version 3.34.1 (*69*) and then log-transformed [e.g., an expression level of 8 (=2^3^) becomes 3, and an expression level of 128 (=2^7^) becomes 7]. Reactive astrocyte marker gene sets were obtained from a previous report (*30*), and the genes contained in the top three astrocyte-related GO terms (GO:0014002, GO:0043615, and GO:0048708) were obtained from QuickGO (www.ebi.ac.uk/QuickGO) (*70*). Genes with standardized values, representing a strength of association of >2.8 between gene and gene set, were used for analysis. The *z* score difference of marker genes for reactive astrocytes between groups were used for transcriptome profiling. For heatmap visualization, log-transformed gene expression was further standardized (average = 0, variance = 1), and the standardized values of *z* scores across treatment groups were visualized as a heatmap using Prism 9 software (GraphPad Software). GO analysis was performed using HOMER version 4.11 (*71*). Differentially expressed genes were defined by *P* < 0.05. The original sequence datasets were deposited to the National Center for Biotechnology Information sequence read archive under accession number GSE201513.

### Quantitative RT-PCR

Total RNA was isolated from the corpus callosum of mouse brains or astrocytes cultured on 35 mm dishes, as described above in the “RNA sequencing” section. Next, cDNA was synthesized from 1 μg or 500 ng of total RNA from the corpus callosum and culture astrocytes, respectively, using ReverTra Ace (Toyobo). Quantitative RT-PCR was performed using the StepOne real-time PCR system (Life Technologies, Carlsbad, CA, USA). The final reaction volume was 10 μl (12.5 ng of cDNA plus THUNDERBIRD SYBR qPCR Mix, Toyobo). The PCR conditions were as follows: heating for 10 min at 95°C, followed by 40 cycles at 95°C for 15 s, 59°C for 30 s, and 72°C for 30 s. The following oligonucleotide primers were used: *Trpa1*, 5′-agg tga ttt tta aaa cat tgc tga g-3′ and 5′-ctc gat aat tga tgt ctc cta gca t-3′; *Gfap*, 5′-agg gcg aag aaa acc gca tc-3′ and 5′-ggt gag cct gta ttg gga ca-3′; *Lif*, 5′-aaa agc tat gtg cgc cta aca-3′ and 5′-gta tgc gac cat ccg ata cag-3′; *Cntf*, 5′-gac ctg act gct ctt atg gaa tct-3′ and 5′-gcc tgg agg ttc tct tgg a-3′; and *18S rRNA*, 5′-gca att att ccc cat gaa cg-3′ and 5′-ggc ctc act aaa cca tcc aa-3′. The amount of *18S rRNA* in samples was used to normalize the mRNA content (the mRNA level was expressed relative to that of the corresponding control).

### Magnetic-activated cell sorting

Whole brains were removed, dissected, and rinsed in Hanks’ balanced salt solution. After centrifuging at 400*g* for 5 min at 4°C and removing the supernatant, enzymatic cell dissociation was performed with collagenase D (5 mg/6 ml per brain in 2% FBS in PBS) and DNase I (60 μl, 10 mg/ml; Sigma-Aldrich) for 1 hour at 37°C with gentle shaking. Dissociated cells were resuspended in 30% Percoll (Sigma-Aldrich) and centrifuged at 700*g* for 30 min at 20°C. The myelin-containing supernatant was discarded, and the pellet was resuspended in cold MACS buffer [containing 1 volume dilution of PBS, 2 mM EDTA, and 0.5% bovine serum albumin (pH 7.2)]. Cell pellets were resuspended in Fc receptor blocking reagent (Miltenyi Biotec) and incubated at 4°C for 10 min. Cells were incubated with anti–ACSA-2 magnetic microbeads (Miltenyi Biotec, catalog no.130-097-678) for 15 min at 4°C. After washing with MACS buffer, centrifuging at 300*g* for 10 min at 4°C, and removing the supernatant, resuspended cells in MACS buffer were applied onto the MS column (Miltenyi Biotec) that had been fixed in the magnetic separator (Miltenyi Biotec). The total RNA of ACSA-2^+^ astrocytes and ACSA-2^−^ flow-through was isolated, and cDNA was synthesized as described above in the “Quantitative RT-PCR” section.

### Assessment of BBB permeability

After anesthesia, the fluorescent tracer sodium fluorescein was injected intravenously (40 mg/kg in saline). After 30 min, brains were dissected, and frozen coronal sections (20 μm thick) were cut as described above in the “Myelin staining” section. Fluorescence was visualized with the FluoView FV10i confocal microscope (Olympus). The positive area of sodium fluorescein in the corpus callosum was measured in a 0.125 mm^2^ field at approximately the bregma +0.7 mm.

### Experimental design and statistical analysis

Statistical analysis was performed using Prism 9 software (GraphPad Software). Briefly, for comparisons between single experimental and control groups, Student’s *t* test or Welch’s *t* test was used. For analysis in Fig. 1L, one sample *t* test was used. For comparisons between multiple experimental groups, one-way analysis of variance (ANOVA) followed by Tukey’s post hoc test or two-way ANOVA with Bonferroni’s or Dunnett’s post hoc test was used accordingly. Differences of *P* < 0.05 were considered statistically significant. Data are provided as means ± SEM.

Each data point represents one sample (a section or extract from specific brain regions, e.g., corpus callosum) of one individual mouse, except for Fig. 1M. The numbers of animals used in each experiment are indicated in the figure legends. The assessor was blinded to the treatment condition.

## Notes

View/request a protocol for this paper from *Bio-protocol*.
