# Supplementary material for: The astrocytic TRPA1 channel mediates an intrinsic protective response to vascular cognitive impairment via LIF production
Source: Sci Adv. 2023 Jul 21;9(29):eadh0102. doi: 10.1126/sciadv.adh0102 (PMC10361588; doi:10.1126/sciadv.adh0102)
Supplement: Supplementary file 1 — Figs. S1 to S8 [file sciadv.adh0102_sm.pdf]

Science Advances
AAAS

Supplementary Materials for

The astrocytic TRPA1 channel mediates an intrinsic protective response to vascular cognitive impairment via LIF production

Masashi Kakae *et al.*

Corresponding author: Hisashi Shirakawa, shirakaw@pharm.kyoto-u.ac.jp

Sci. Adv. **9**, eadh0102 (2023)
DOI: 10.1126/sciadv.adh0102

This PDF file includes:

Figs. S1 to S8

Supplementary Figures & Legends

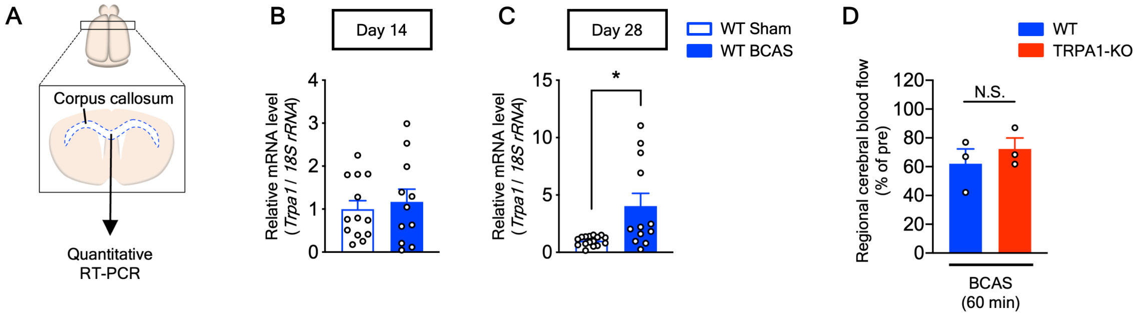

Kakae et al.,

Fig. S1. Bilateral common carotid artery stenosis (BCAS) increases *Trpa1* mRNA expression in the corpus callosum on day 28.

(A) A schematic of quantitative RT-PCR of the corpus callosum. (B and C) *Trpa1* mRNA expression in the corpus callosum on days 14 (B) and 28 (C). (D) Regional cerebral blood flow in the middle cerebral artery territory compared with the baseline at 60 min after operation. Values are mean ± SEM. (B) n = 11–13; (C) n = 12–16; (D) n = 3. *$p < 0.05$ for two-tailed unpaired Welch's t-test (C).

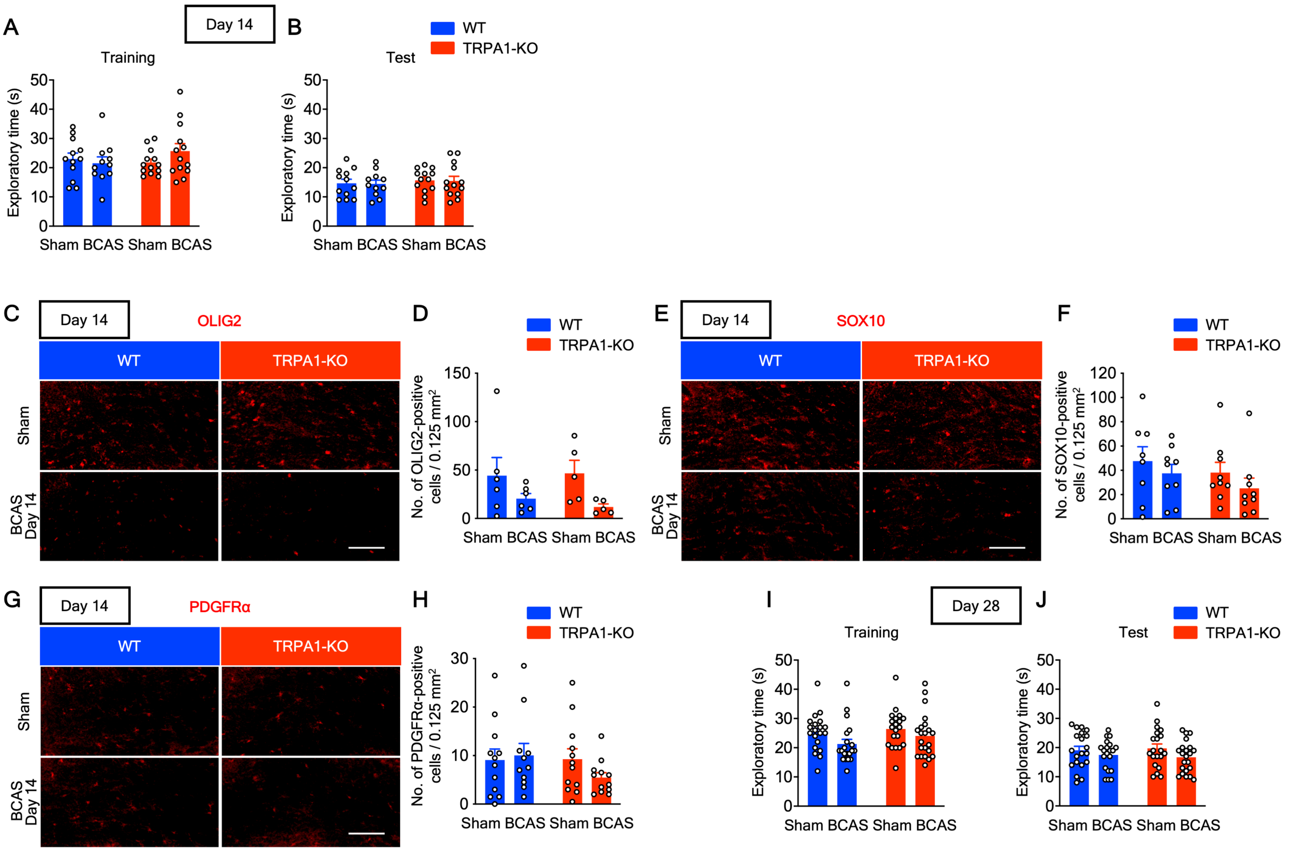

Kakae et al.,

Fig. S2. Exploratory behaviors are similar between the groups in the novel object recognition test (NORT) and bilateral common carotid artery stenosis (BCAS) does not induce loss of immature oligodendrocyte lineage cells.

(**A**, **B**, **I**, and **J**) Total exploratory times during the training (A and I) and test sessions (B and J) on days 14 (A and B) and 28 (I and J). (**C** to **H**) Representative images of immunostaining with anti-OLIG2 (C), SOX10 (E), or PDGFRα (G) antibody in the corpus callosum and summarized data for the number of OLIG2- (D), SOX10- (F), or PDGFRα- (H) positive cells on day 14. Values are mean ± SEM. Scale bars, 100 μm (C, E, and G). (A and B) n = 11–13; (D) n = 5–6; (F) n = 8–9; (H) n =11–12; (I and J) n = 20–22.

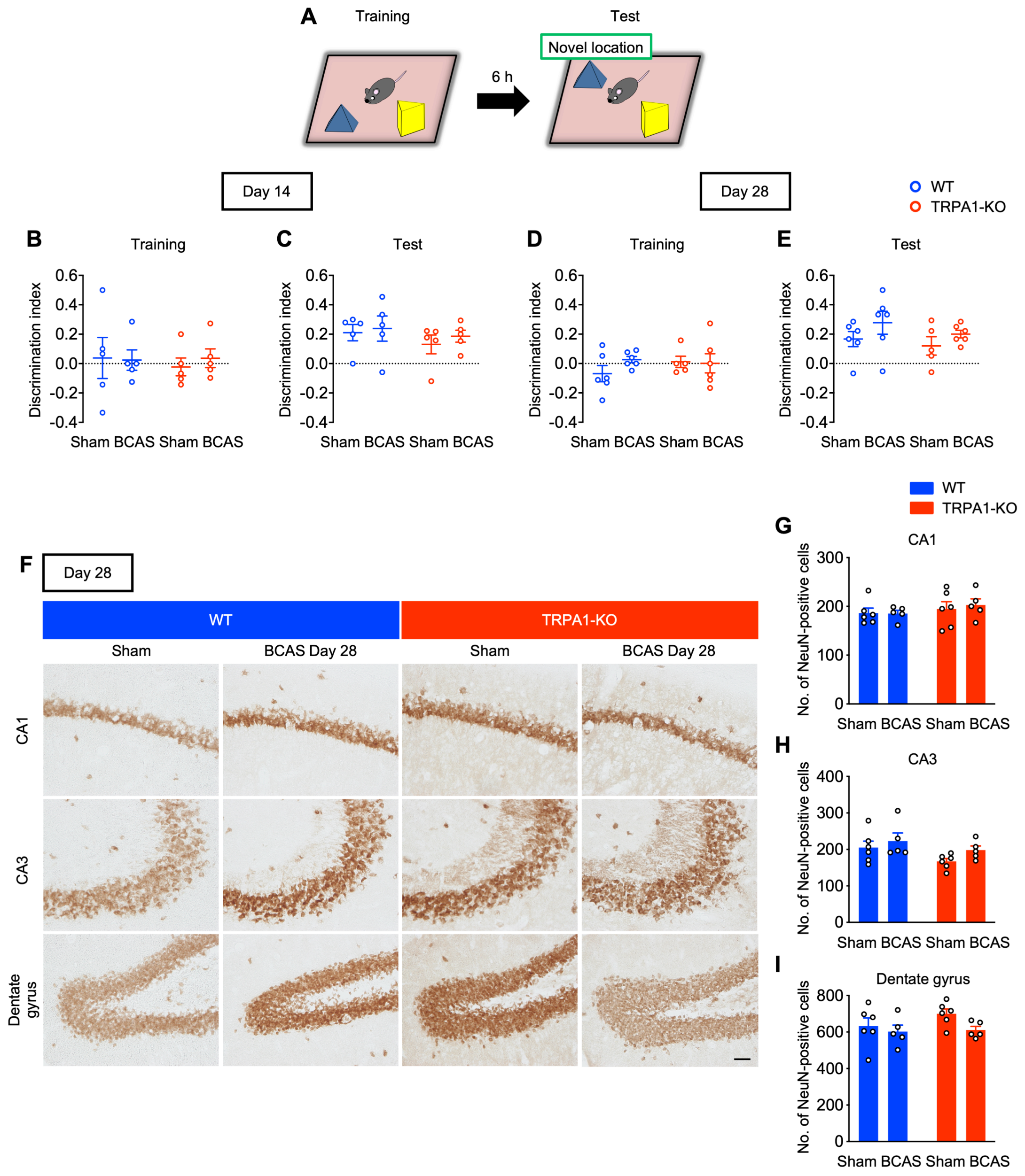

Kakae et al.,

Fig. S3. Bilateral common carotid artery stenosis (BCAS) does not induce spatial memory dysfunction or neuronal damage in the hippocampus.

(**A**) A schematic of the novel location recognition test (NLRT). (**B** to **E**) Discrimination indexes (DI) for exploring the blue quadrangular object during the training sessions (B and D) and the relocated object, i.e., the "novel location," during the test sessions (C and E) on days 14 (B and C) and 28 (D and E). (**F** to **I**) Representative images of immunostaining with anti-NeuN antibody in the hippocampus (F), and summarized data for the number of NeuN-positive cells in CA1 (G), CA3 (H), and dentate gyrus (I) on day 28. Values are mean ± SEM. Scale bar, 10 µm. (B and C) $n = 5$; (D, E, G, H, and I) $n = 5–6$.

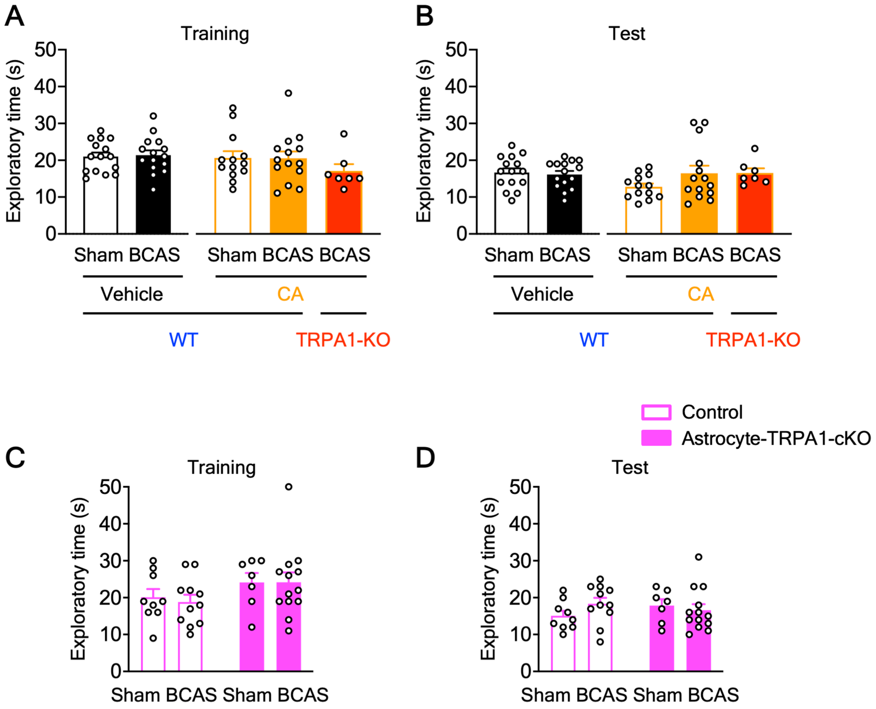

Kakae et al.,

Fig. S4. Exploratory behaviors are similar between the groups in the novel object recognition test (NORT) in cinnamaldehyde (CA) administration experiments, or in astrocyte-specific TRPA1 deficiency experiments.

(**A** and **B**) Total exploratory times during the training (A) and test sessions (B) on postoperative day 28 after daily administration with CA. (**C** and **D**) Total exploratory times during the training (C) and test sessions (D) on day 14 in astrocyte-TRPA1-cKO mice. Values are mean ± SEM. (A and B) n = 15 (vehicle), n = 7–14 (CA); (C and D) n = 7–13.

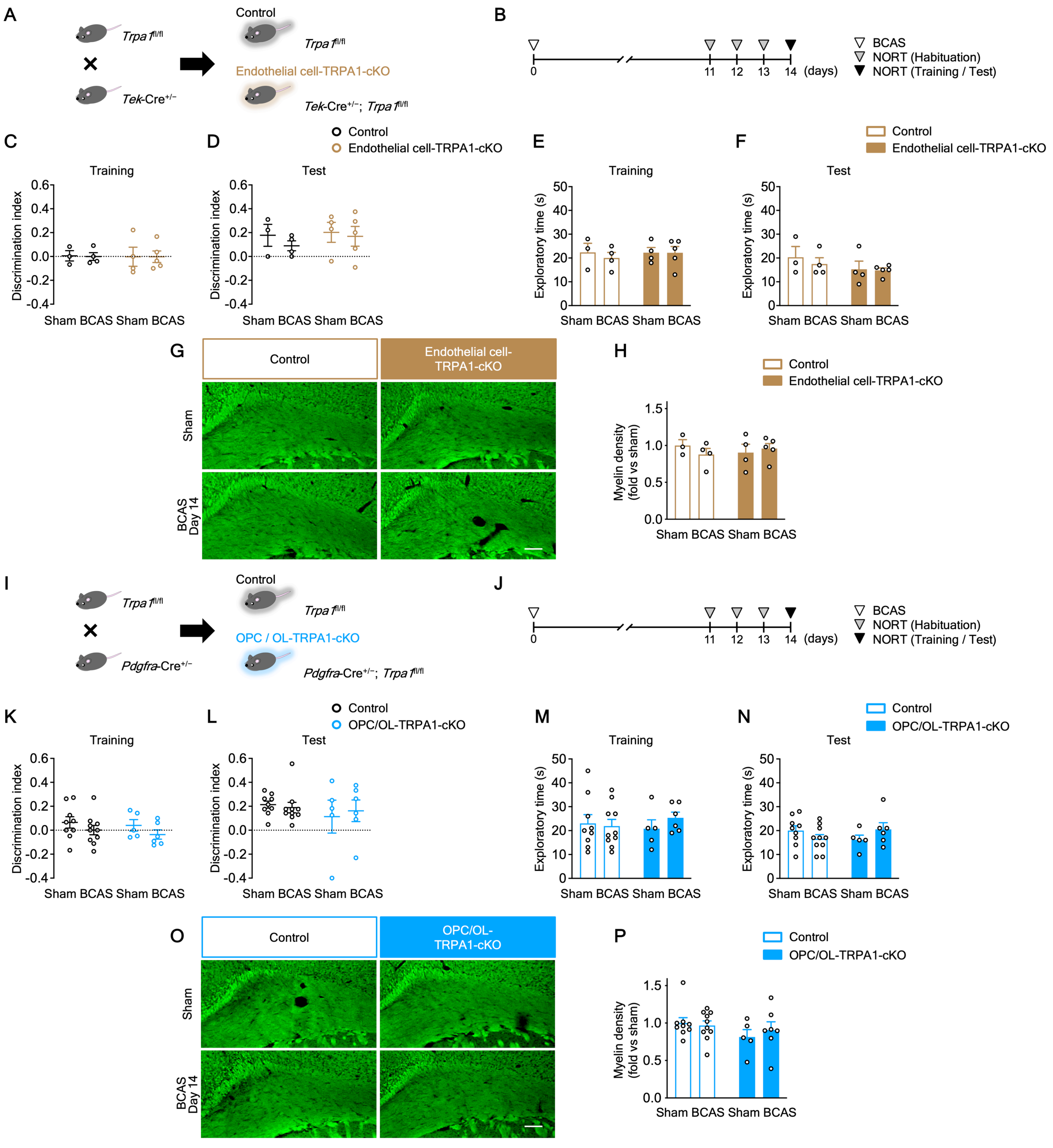

Kakae et al.,

Fig. S5. Endothelial cell-TRPA1-cKO and oligodendrocyte precursor cell (OPC)/oligodendrocyte (OL)-TRPA1-cKO do not show bilateral common carotid artery stenosis (BCAS)-induced cognitive impairment or white matter injury on day 14.

(**A** and **I**) A schematic for obtaining endothelial cell-TRPA1-cKO (A) or OPC/OL-TRPA1-cKO (I) and control mice. (**B** and **J**) The experimental time courses for the novel object recognition test (NORT). (**C**, **D**, **K**,

and **L**) Discrimination indexes for exploring the blue quadrangular object during the training sessions (C and K) and the wooden ball, i.e., “novel object,” during the test sessions (D and L) on day 14. (**E**, **F**, **M**, and **N**) Total exploratory times during the training (E and M) and test sessions (F and N) on day 14 in endothelial cell-TRPA1-cKO (E and F) and OPC/OL-TRPA1-cKO (M and N) mice. (**G**, **H**, **O**, and **P**) Representative images of myelin staining in the corpus callosum (G and O) and summarized data for relative myelin density (H and P) on day 14 in endothelial cell-TRPA1-cKO (G and H) and OPC/OL-TRPA1-cKO (O and P) mice. Values are mean ± SEM. Scale bars, 100 μm. (C, D, E, F, and H) n = 3–5; (K, L, M, N, and P) n = 5–10.

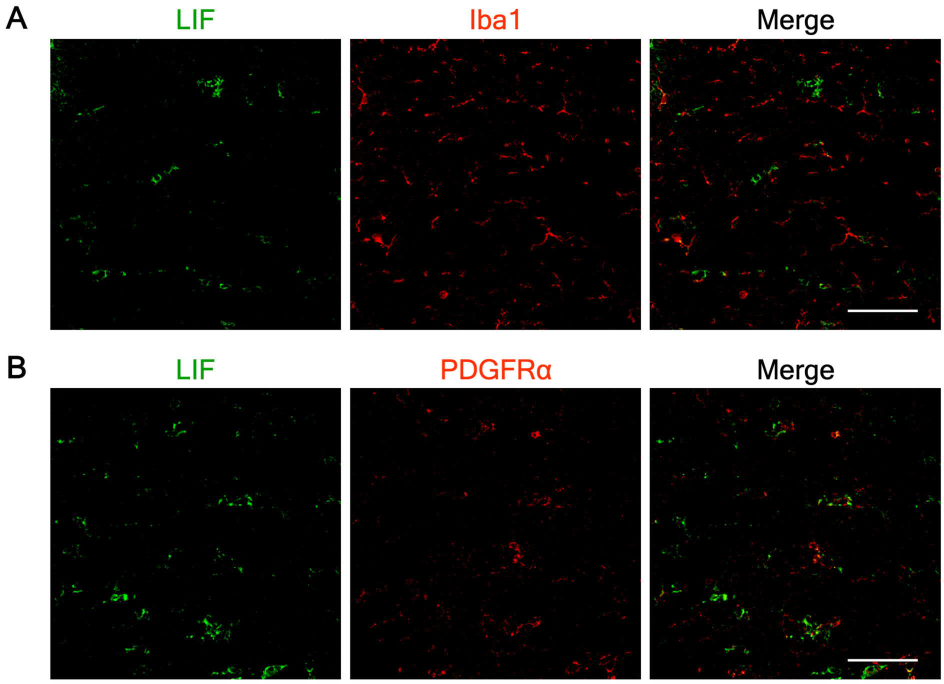

Kakae et al.,

Fig. S6. The cellular sources of LIF induced by bilateral common carotid artery stenosis (BCAS) are not microglia and oligodendrocyte precursor cells (OPCs).

(**A** and **B**) Representative images of immunostaining with anti-LIF antibody (green) and anti-Iba1 (A) or PDGFRα (B) antibody (red) in the corpus callosum of BCAS-operated WT mice on day 14. Scale bars, 50 μm.

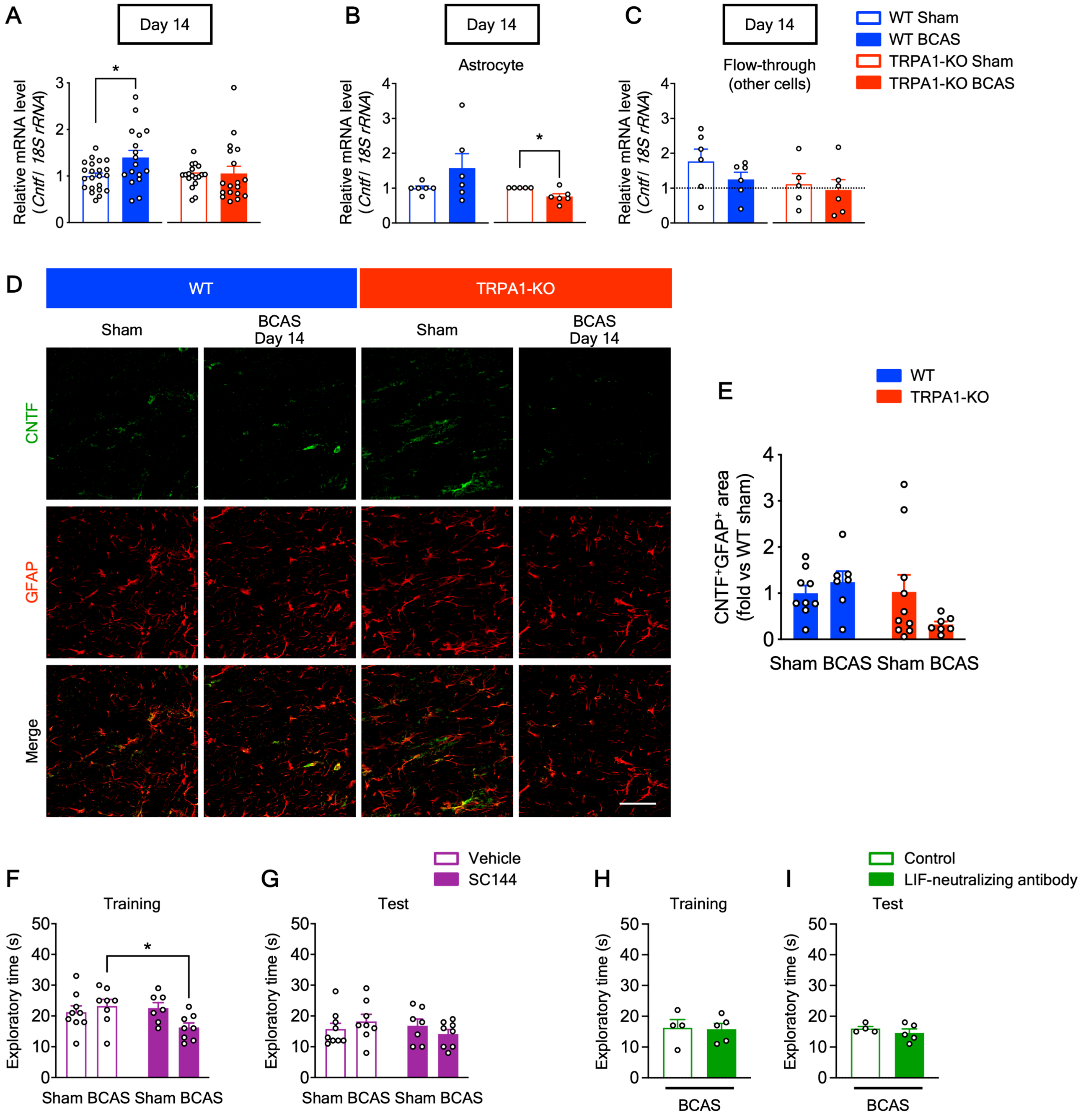

Kakae et al.,

Fig. S7. Bilateral common carotid artery stenosis (BCAS) does not increase ciliary neurotrophic factor (*Cntf*) mRNA expression in astrocytes.

(**A** to **C**) *Cntf* mRNA expression in the corpus callosum (A), isolated astrocytes (B), and flow-through (C), determined by quantitative RT-PCR on day 14. (**D** and **E**) Representative images of immunostaining with anti-CNTF antibody (green) and anti-GFAP antibody (red) in the corpus callosum (D), and summarized data for the percentage of CNTF-/GFAP-double-positive surface areas (E) on day 14. (**F** to **I**) Total exploratory

times during the training (F and H) and test session (G and I) on postoperative day 14. Values are mean ± SEM. Scale bar, 50 μm. (A) n = 17–22 (WT), n = 18 (TRPA1-KO); (B and C) n = 6 (WT), n = 5–6 (TRPA1-KO); **(**E) n = 7–10; (F and G) n = 7–9; (H and I) n = 4–5. *$p < 0.05$ for two-tailed unpaired Welch's t-test (A and B) and two-way ANOVA with Bonferroni's *post hoc* test (F).

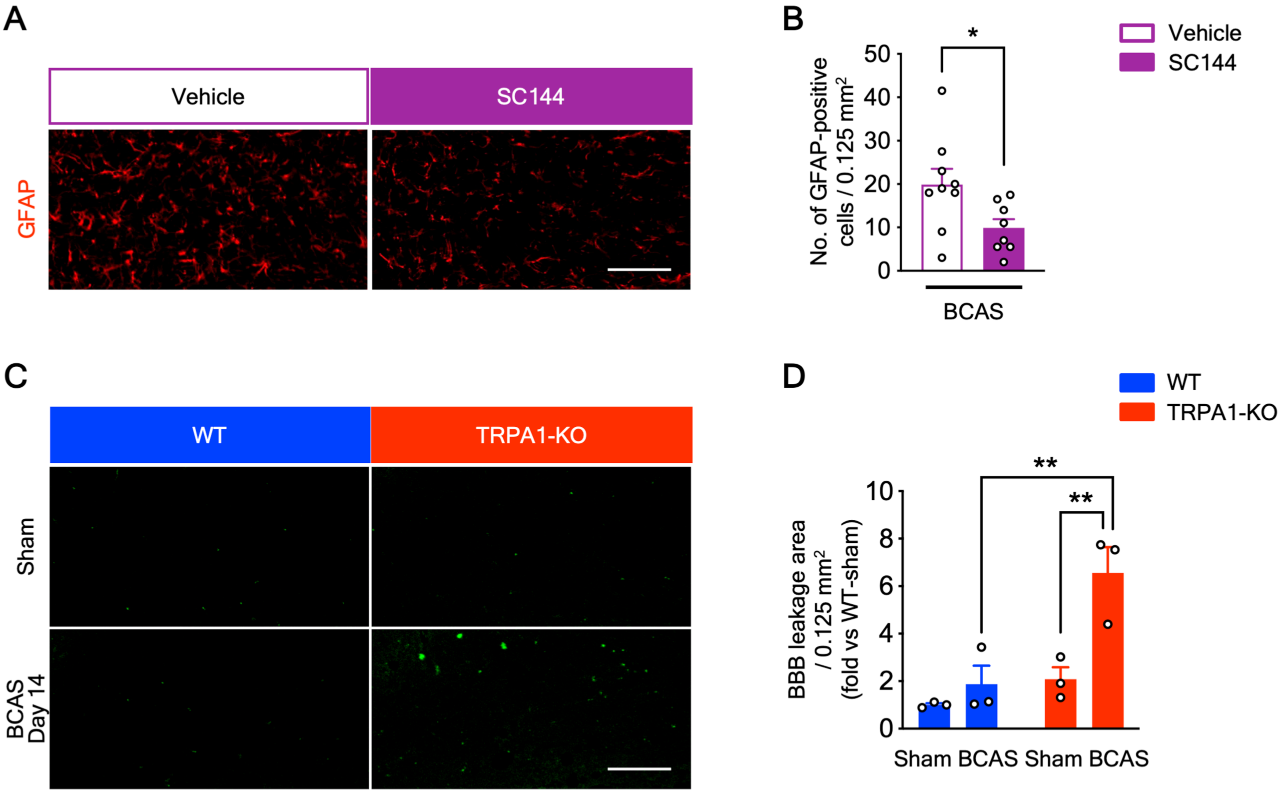

Kakae et al.,

Fig. S8. A leukemia inhibitory factor (LIF) signaling inhibitor, SC144, suppresses the increase of GFAP-positive astrocytes and bilateral common carotid artery stenosis (BCAS) increases the permeability of the blood-brain barrier on day 14 in TRPA1-KO but not WT mice.

(**A** and **B**) Representative images of immunostaining with anti-GFAP antibody in the corpus callosum (A) and summarized data for the number of GFAP-positive cells (B) on postoperative day 14 in the experiments with SC144. (**C** and **D**) Representative images (C) and summarized data (D) for the percentage of sodium fluoresceine-positive surface areas in the corpus callosum on day 14. Values are mean ± SEM. Scale bars, 100 μm. (B) n = 8–9; (D) n = 3. *$p < 0.05$ for two-tailed unpaired Student's t-test (B); **$p < 0.01$ for two-way ANOVA with Bonferroni's *post hoc* test (D).
